# Deletion of *Snai2* and *Snai3* Results in Impaired Physical Development Compounded by Lymphocyte Deficiency

**DOI:** 10.1371/journal.pone.0069216

**Published:** 2013-07-16

**Authors:** Peter D. Pioli, Timothy J. Dahlem, Janis J. Weis, John H. Weis

**Affiliations:** The Division of Cell Biology and Immunology, Department of Pathology, University of Utah School of Medicine, Salt Lake City, Utah, United States of America; Centro de Investigación en Medicina Aplicada (CIMA), Spain

## Abstract

The Snail family of transcriptional regulators consists of three highly conserved members. These proteins regulate (repress) transcription via the recruitment of histone deacetylases to target gene promoters that possess the appropriate E-box binding sequences. Murine *Snai1* is required for mouse development while *Snai2* deficient animals survive with some anomalies. Less is known about the third member of the family, *Snai3*. To investigate the function of *Snai3*, we generated a conditional knockin mouse. Utilizing *Cre*-mediated deletion to facilitate the ablation of *Snai3* in T cells or the entire animal, we found little to no effect of the loss of *Snai3* in the entire animal or in T cell lineages. This finding provided the hypothesis that absence of Snai3 was mitigated, in part, by the presence of Snai2. To test this hypothesis we created *Snai2/Snai3* double deficient mice. The developmental consequences of lacking both of these proteins was manifested in stunted growth, a paucity of offspring including a dramatic deficiency of female mice, and impaired immune cell development within the lymphoid lineages.

## Introduction

Snail transcription factors (TF) comprise a highly conserved family consisting of three members: *Snai1* (*Snail*), *Snai2* (*Slug*), and *Snai3* (*Smuc*) [1,2]. *Snai1* was first discovered in *Drosophila melanogaster* [3] and all 3 members have been identified in organisms ranging from *D. melanogaster* and *Caenorhabditis elegans* to *Mus musculus* and *Homo sapiens* [4]. The encoded proteins share high sequence homology and range from 30–37 kilodaltons (kD) in size. All members share two characteristic features: an amino terminal SNAG (Snail and Gfi-1) domain and zinc finger DNA-binding domains (DBDs) (five DBD domains for Snai2 and Snai3 and four for Snai1) in the carboxy terminus [4]. These transcription factors recognize the consensus E-box sequence, CANNTG [5] preferentially binding to E-boxes that possess GC-rich central di-nucleotides as opposed to, for example, MyoD that prefers to bind to E-box sites enriched for AT central di-nucleotides [5]. While the DBDs determine binding specificity, it is the SNAG domain that imparts functionality to these proteins. Through this domain, Snail TFs interact with various histone deacetylases (HDACs) resulting in the silencing of target gene expression [6,7].

Previously, the roles of Snail members in embryonic and muscle development have been defined. Germline deletion of *Snai1* is an embryonic lethal due to gastrulation defects [8,9]. All three Snail members have been shown to negatively regulate muscle differentiation by competing for E-box binding with other myogenic regulatory factors (MRFs) [5,10]. Additionally the members of the Snail family have been linked to epithelial-mesenchymal transition, the migration of neural crest cells and generation of neural tubes, the regulation of E-cadherin which is linked to the progression of cancer metastasis, and controlling the response to apoptosis initiators (for reviews, see 11,12). For example, *Snai2* deficient animals are more sensitive to total body γ irradiation than WT [13], and *Snai2* deficient hematopoietic progenitor cells demonstrate enhanced levels of apoptosis following radiation-induced DNA damage than WT cells [13,14]. A later study described the role of Snai2 in antagonizing p53-mediated apoptosis in hematopoietic precursor cells by inhibiting Puma (Bbc3) [15]. Snai2 also has a variety of functions in skin development, response to skin insults (sunburn, wound healing, skin cancer) and hair growth [16,17].

The role of the Snail proteins in immune cell development is less defined. A report by Inukai et al. demonstrated that *Snai2* over-expression in IL-3-dependent Baf cells (pro-B cell line) overcame the apoptotic stimuli induced by IL-3 withdrawal [18]. Perez-Losada et al. reported that germline deletion of *Snai2* resulted in diminished CD4^+^CD8^+^ double positive (DP) T cell cells in the thymus which skewed the population to enhanced numbers of CD4^+^ single positive (SP) thymocytes, similar to that found in animals with deficient c-kit signaling [19]. This report further linked *Snai2* expression to c-kit pathways, demonstrating erythroid development defects and pigmentation anomalies in the *Snai2* deficient animals, but normal B cell and myeloid cell development. Bone marrow chimera models demonstrated that such defects were intrinsic to the stem cell [19]. Others have also reported that the numbers of T and B cells, the mitogenic responses of splenic and thymic lymphocytes and circulating blood cell counts in *Snai2KO* animals were equivalent to WT [13]. Snai2 does appear to have fundamental functions in early steps of hematopoiesis. The expression of the gene is apparent in both long term and short tem repopulating hematopoietic stems cells, in common lymphoid and myeloid precursor populations and precursors in the granulocyte, megakaryocyte and erythrocyte lineages [13]. Interestingly hematopoietic stem cell precursors that lack Snai2 show a heightened ability to repopulate the animal following 5-FU treatment, compared to WT, suggesting that Snai2 functions to negatively regulate the self-renewal division of such cells [20].

We have shown that the over-expression of *Snai3* in hematopoietic stem cell lineages resulted in the loss of mature lymphocytes and the enhanced development of cells of the myeloid lineage [21] suggesting that lymphoid/myeloid fate decisions are controlled, in part, by E-box binding proteins with a predilection for GC-rich central di-nucleotides. In this study, we took the opposite approach and attempted to define the phenotypes of mice lacking *Snai3* in T cell lineages (due to the high level of expression of *Snai3* in developing T cells) and the entire animal, and subsequently the phenotype of mice lacking functional *Snai2* and *Snai3* genes. *Snai3* is highly expressed in T cell lineages (both DP cells of the thymus and CD8^+^ cells in the periphery) however deletion of this gene in either T cell lineages or the entire animal had little effect upon animal development or T cell lineages/functions. Since *Snai2* had previously been shown to alter thymocyte development, we generated *Snai2/Snai3* double KO (DKO) animals to test for functional complementation of the two gene products. Mice lacking both of these genes demonstrated much greater physical anomalies than single *Snai2KO* or *Snai3KO* animals including severe running and the dramatic lack of female offspring. Effects to cells of the immune system upon *Snai2/Snai3* deletion included the altered development of double positive (CD4^+^CD8^+^) thymocytes and a significant impairment of B cell development in the bone marrow. Overall, our data demonstrate a significant physiological role for *Snai2* and *Snai3* within cells of the immune system.

## Results

### Snai3 is not required for thymocyte development

**Figure 1:**
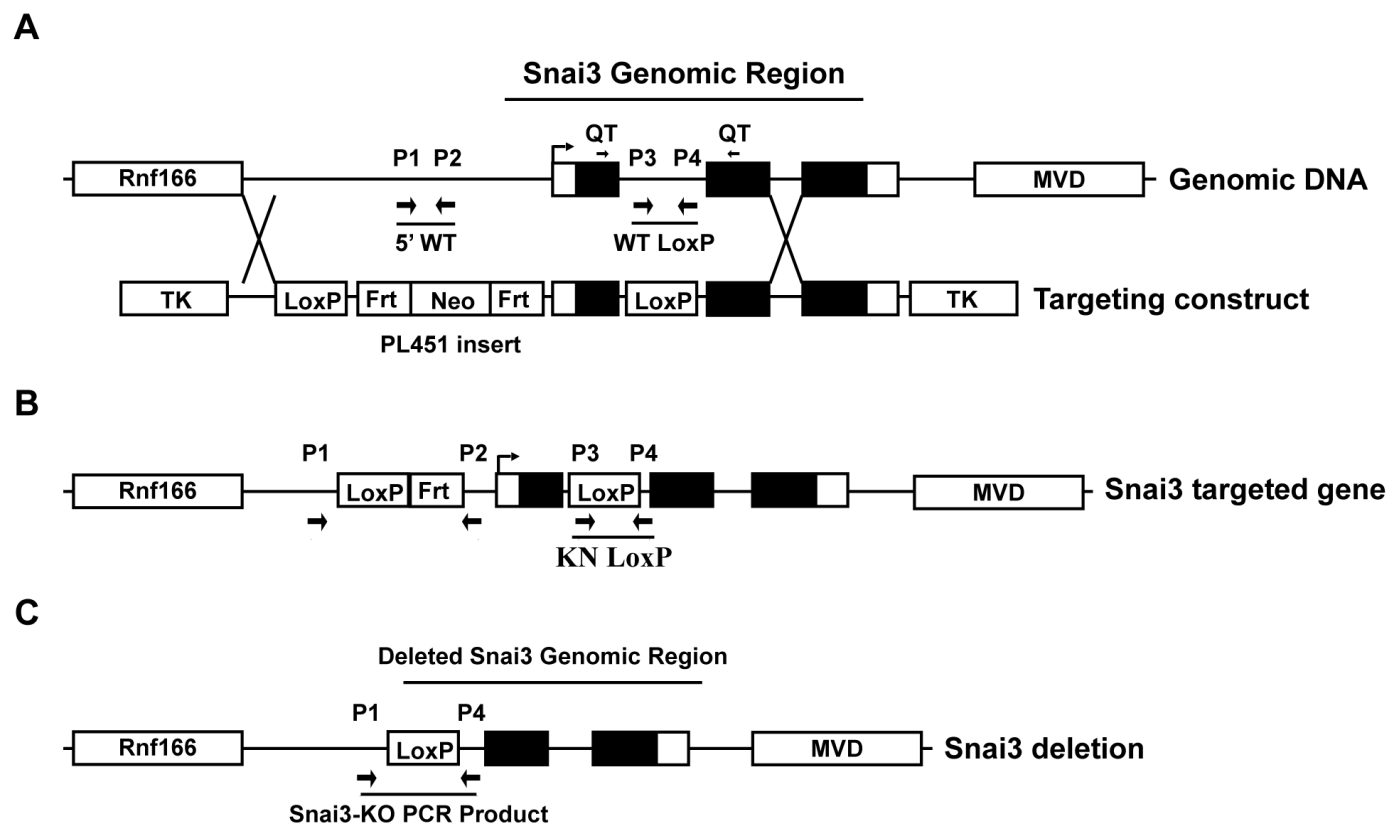
Conditional *Snai3* deletion strategy. (A) The *Snai3* WT genomic DNA region contains the *Rnf166* gene 5 kb upstream and the *MVD* gene 10 kb downstream. The *Snai3* gene consists of 5’ and 3’ untranslated regions (white boxes), three exons (black boxes), and two introns. Arrow marks the transcriptional start site (TSS). Primers used for genotyping mice and for QT-PCR of *Snai3* transcript are labeled as P1-P4 and QT, respectfully, and listed in Supplemental Table 1. The Targeting Construct had a LoxP site inserted into the PacI site of intron one and the PL451 cassette inserted into the SalI site 2kb upstream of the TSS. (B) Homologous recombination created the *Snai3* targeted genome containing unique 5’ PL451 and Knockin LoxP PCR products. The Neomycin (Neo) cassette was deleted via FLP-mediated recombination of Frt sites. (C) Cre recombinase activity deleted the *Snai3* genomic region and created a new PCR product by bringing together primers P1 and P4, which are normally 6kb apart and unable to make a PCR product. Figure is not to scale.

The gene encoding the mouse Snai3 transcription factor is highly expressed in three tissue samples of the animal: skeletal muscle, cardiac muscle and the thymus (Figure S1 in File S1) [10,22]. Expression was also noted in the kidney, liver and spleen, all organs with significant numbers of blood cells. These data suggested that germline deletion of *Snai3* might result in an embryonic lethality (similar to that of a *Snai1* deletion) due to defects in skeletal or cardiac muscle development. Since we were primarily interested in the role of Snai3 in the development of bone marrow lineages, including T cells, we generated a *Snai3* knockout (*Snai3KO*) construct amenable to *Cre*-mediated deletion (Figure 1). This construct resulted in the placement of LoxP recombination sites 5’ and 3’ of the first exon containing the initiating ATG and encoding the SNAG domain.

Once obtained, the *Snai3KO* mouse was bred to the *Lck-Cre* animal, which would delete *Snai3* specifically in T cells at the double negative stage in thymocyte development [23,24]. Successful deletion of the *Snai3* gene in T cell but not B cell lineages was confirmed by RT-PCR of *Snai3* transcripts (Figure S2 in File S1). FACS analysis of samples obtained from the *Snai3* conditional deletion animals indicated there were no alterations in steady state T cell populations in the thymus, spleen or blood (Figure S3 in File S1) suggesting that if Snai3 was essential for the development of T cells, its action was prior to the expression of *Lck*. To test this possibility, we bred the *Snai3KO* mouse with a *Hprt*-*Cre* deleter strain which resulted in the germline deletion of *Snai3* [25]. *Snai3* homozygous deleted mice were viable and displayed no apparent developmental defects when compared to WT mice. This included growth rates, offspring sex ratios and fertility. Analysis of transcript (Figure S4 in File S1) and protein production (Figure S5 in File S1) demonstrated a loss of the *Snai3* gene products in the germline *Snai3KO* animals. Analysis of T cells in the thymus, and T and B cells in the periphery was also the same as WT (data not shown) suggesting that Snai3 alone is not essential for the development of such cells.

### Differential expression of *Snai1*, *Snai2* and *Snai3*

The absence of any demonstrable phenotype in mice lacking Snai3 suggested that its function may be irrelevant to the animal, or that its absence may be complemented by that of the other Snail family members. The *Snai3* coding sequence has been highly conserved over vertebrate evolution suggesting it does serve a defined function(s) [26]. Alternatively many instances of transcription factor functional redundancy have been demonstrated. MEF2 family members have been shown to display partially overlapping functions in neuronal development [27]. More relevantly, both Snai1 and Snai2 have exhibited migratory and anti-apoptotic functions when studied in cancer models [11,28–30]. Additionally, all three Snail family members have been shown to inhibit myoblast differentiation *in vitro* [5,10] and all three possess very conserved SNAG and DNA binding protein domains [26]. Animals require the Snai1 protein in that the loss of *Snai1* results in an embryonic lethality [8,9]. Animals lacking *Snai2* are born viable but demonstrate decreased growth rate, skewing of immature T cell populations, eyelid defects and deficiencies in erythropoiesis and fertility [13,19,31].

**Figure 2:**
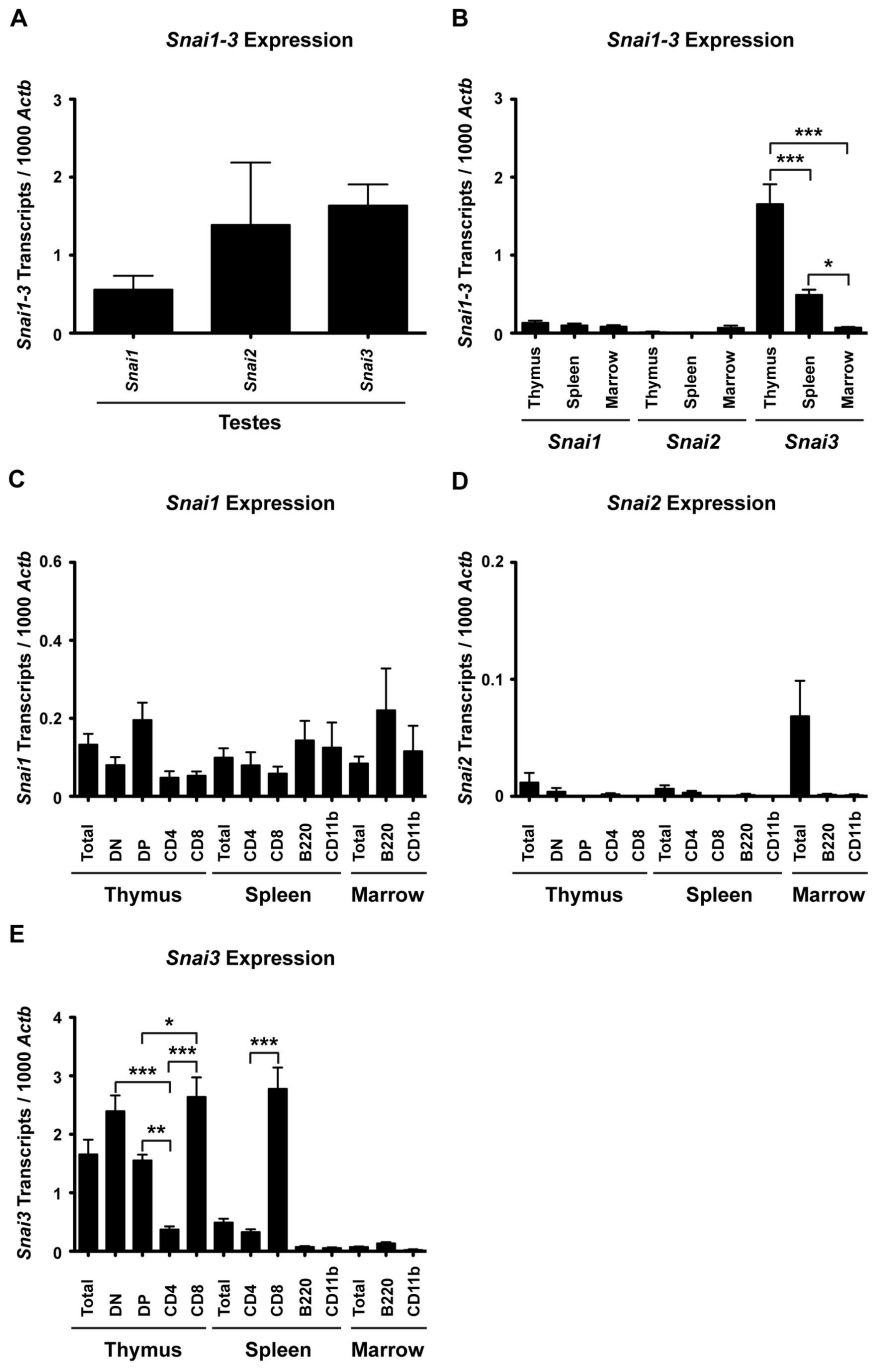
*Snai1-3* transcripts are differentially expressed in primary and secondary lymphoid organs of wild type mice. RNA was isolated and cDNA synthesized as described in the *Materials*. Quantitative real-time RT-PCR was performed via LightCycler as described in the *Materials*. (A) cDNA from testes served as a positive control for all RT-PCR analysis. (B) *Snai1-3* transcript analysis of cDNA generated from total thymus, spleen, and bone marrow. (C–E) RT-PCR of specific cell subsets demonstrating the distribution patterns of *Snai1* (C), *Snai2* (D), and *Snai3* (E) expression. Three mice were analyzed for the testes. At least 4 mice were analyzed per immune cell type. Levels of *Snai1-3* transcripts are relative to 1000 *Actb* transcripts per each individual sample. Data are represented by the mean ± SEM. One-way ANOVA with Bonferroni post hoc test: * p < 0.05, ** p < 0.01, *** p < 0.001.

To determine if either Snai1 or Snai2 could potentially complement Snai3, we first evaluated their expression in defined tissue and cell types using quantitative RT-PCR (Figure 2). We confirmed the specificity of the oligonucleotide sets using testes mRNA which is highly enriched for transcripts from all three Snail family members (Figure 2A). Whole tissues (thymus, spleen and total marrow cells) were also analyzed for *Snai1*, *Snai2* and *Snai3* expression (Figure 2B). Due to differences in amplification efficiencies it is not possible to directly compare the absolute numbers of gene specific transcripts to each other but clearly *Snai3* is expressed in thymus, spleen and marrow samples as is *Snai1* while *Snai2* is most highly expressed in marrow cells. RNA was then obtained from FACS sorted thymus, spleen and marrow cell populations (see *Materials*) and analyzed. While *Snai1* was expressed in all populations analyzed (Figure 2C), there was a clear enrichment of transcripts in the DP thymocyte population relative to all other thymic cell types examined. Additionally, *Snai*1 expression was evident in mature T cells, B cells and myeloid cells of the spleen and in immature B cell and myeloid lineages of the bone marrow. The expression of *Snai2* was detectable in the most immature of T cell subsets of the thymus (thymus double negative, DN, populations) and CD4^+^ T cells in the spleen but was most evident in immature precursor and stromal cells of the marrow (B220^-^ or CD11b^-^ cells) (Figure 2D). Cells in these populations include uncommitted and committed progenitor cells with the potential to generate myeloid and lymphoid cell lineages.

*Snai3* expression was apparent in the DN and DP thymocyte populations, and maintained in the CD8^+^ SP cells of the thymus and spleen (Figure 2E). The loss of *Snai3* in T cells after commitment to the CD4^+^ SP lineage is evident in both maturing cells in the thymus and mature peripheral cells in the spleen, potentially suggesting a role for Snai3 in defining the CD4^+^ versus CD8^+^ fate decision [32,33]. However, as described above, total deletion of *Snai3* had no effect on CD4^+^ or CD8^+^ SP populations of the thymus or spleen. In bone marrow, *Snai3* was expressed at increased levels in the B cell lineage relative to myeloid cells (Figure 2E).

**Figure 3:**
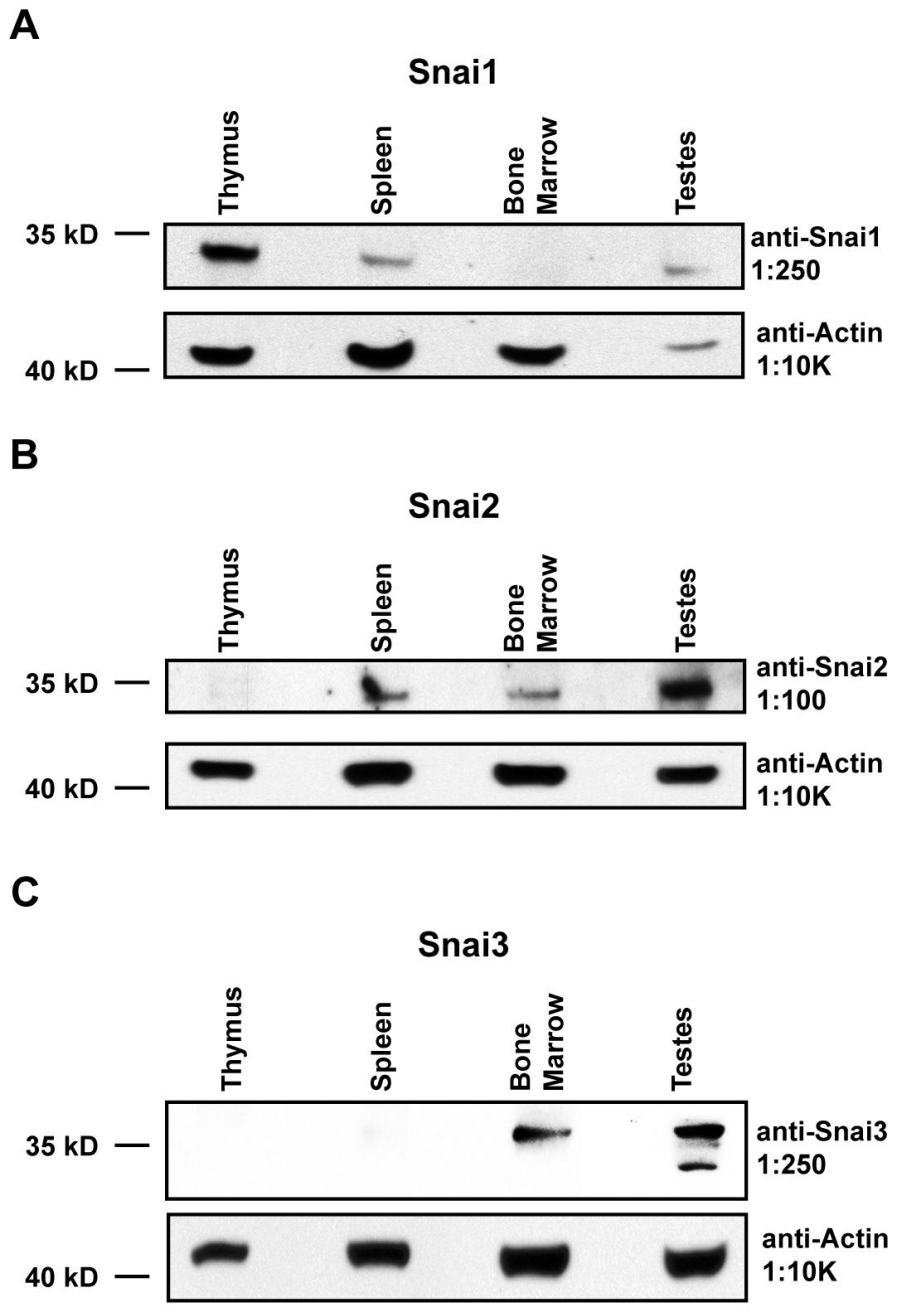
Snai1-3 protein expression suggests post-translational regulation. Whole cell lysates were generated from total thymus, spleen, bone marrow, and testes. Samples were subjected to immunoblot analysis as described in the *Materials*. (A–C) Samples were probed with primary antibodies specific for Snai1 (A), Snai2 (B), and Snai3 (C). Blots were probed for β-actin to verify equal protein loading. Representative blots are shown but similar results were generated for two independent mice.

To confirm whether protein expression paralleled transcript data, whole cell lysates were generated from total thymus, spleen, bone marrow, and testes as a positive control. Lysates were electrophoresed and blotted (Western blot: see *Materials*). As shown in Figure 3A, Snai1 (~30 kD) protein levels were highest in the thymus, lower in the spleen and undetectable in the bone marrow although transcript levels would have predicted that thymus and marrow would have provided nearly equivalent protein quantities. Snai2 (~34 kD) protein was detectable in bone marrow and spleen but not thymus (Figure 3B). [The specificity of the anti-Snai2 antibody was confirmed by comparing the presence of the protein in testes obtained from WT and animals lacking both Snai2 and Snai3 (Figure S6A,B in File S1)]. Post-transcriptional/translational regulation may be controlling Snai1 and/or Snai2 protein levels. For example, both Snai1 and Snai2 are targeted for degradation via GSK3β and MDM2 dependent mechanisms, respectively [34,35]. Additionally *Snai2* transcripts were recently described as targets of miR-206 [5] and *Snai1* transcripts as targets of miR-30a [36] the result of which can destabilize the gene transcripts and block translation.

The Snai3 protein (~36 kD) was detectable in the testes and less so in the marrow but not found in either spleen or thymus samples (Figure 3C). These data are also in contrast to the transcript data in that thymus showed much higher levels of *Snai3* transcripts than either spleen (primarily from CD8^+^ SP T cells) or marrow. The specificity of the anti-Snai3 antibody was confirmed by comparing the presence of the protein in bone marrow obtained from WT and *Snai3KO* animals (Figure S5 in File S1). The production of Snai3 protein may also be affected by miRNA functions in that the mouse *Snai3* transcript also possesses a seed sequence for binding to miR-182-3p (data not shown).

### Animals lacking Snai2 and Snai3 demonstrate more profound anatomical deficiencies than singe KO animals

The expression data described above suggested that the expression of *Snai1*, *Snai2* and *Snai3* may overlap in specific cell types/tissues and that a different family member may functionally complement the deficiency of the Snai3 protein in the *Snai3KO* mouse. To test for this possibility, we created a double KO animal lacking both *Snai2* and *Snai3* (DKO). As mentioned above, germline deletion of *Snai1* results in embryonic lethality. In contrast, single germline deletions of either *Snai2* [13,31] or *Snai3* are viable although *Snai2* deficient males are described as having decreased fertility due to testicular atrophy [19].

**Figure 4:**
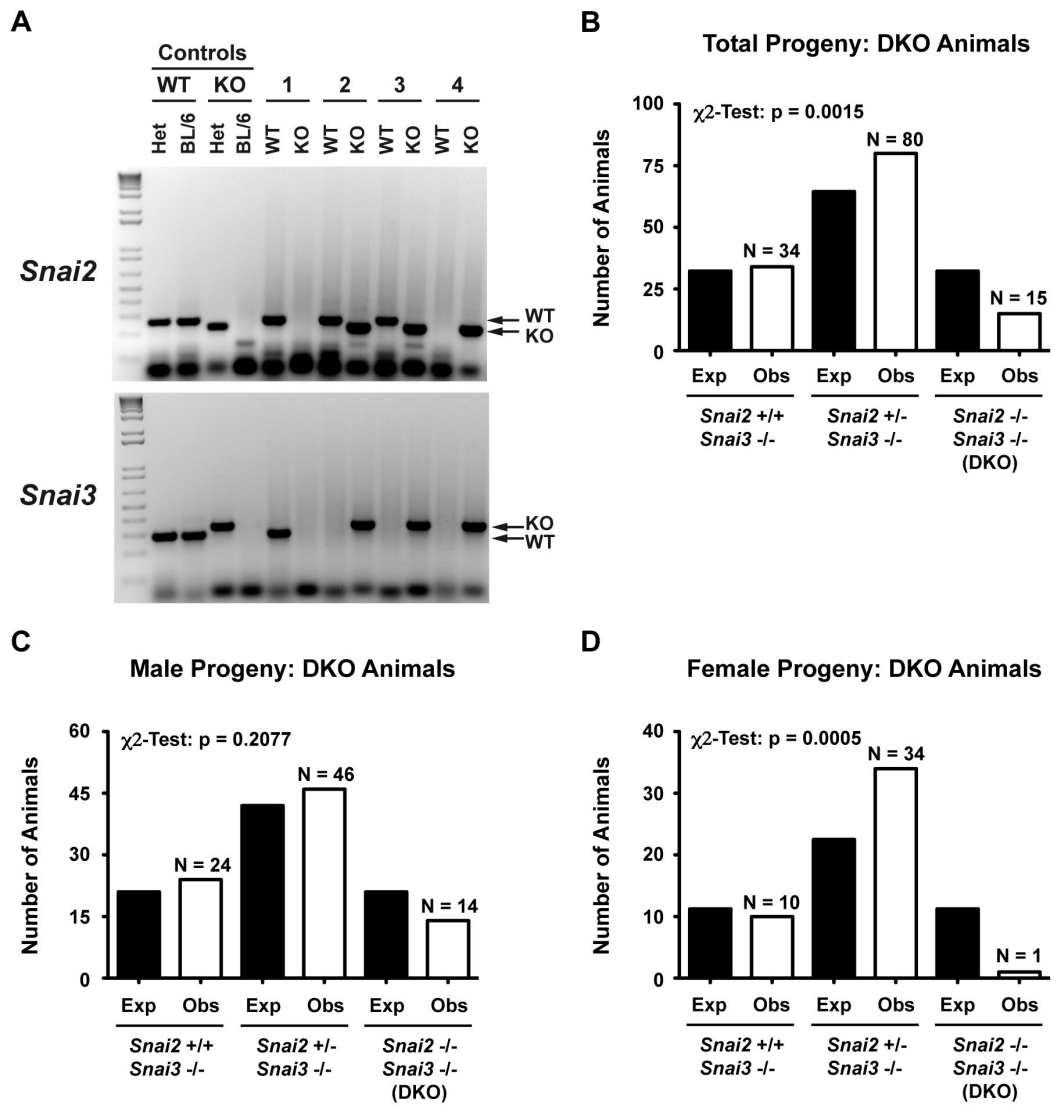
Generation and Mendelian analysis of *Snai2* and *Snai3* double knockout (DKO) mice. (A) Representative 2% agarose gels demonstrating genotyping of *Snai2* and *Snai3*. WT and KO refer to primer sets specific for the wild type or knocked out allele. 1-4 refer to 4 separate experimental mice. Het = heterozygous control DNA, BL/6 = wild type control DNA (B) Analysis of all progeny derived from mating *Snai2* ^*+/-*^ *Snai3* ^*-/-*^ parents. Black bars indicate expected numbers while the open bars indicate actual progeny obtained per genotype. (C–D) Distribution of male (C) and female (D) DKO animals generated from *Snai2* ^*+/-*^ *Snai3* ^*-/-*^ parents. Animal numbers per group are represented by the “N” values (open bars) while predicted numbers based upon the numbers of males and females generated in the DKO colony are shown in the black bars. More total males of all three genotypes (about 2:1) were generated in this mouse mating scheme compared to females with all of the combined genotypes. χ^2^-test analysis for panels B, C, D demonstrates significance between predicted progeny genotypes and those obtained.

In order to create a *Snai2/3DKO* we first bred the *Snai2KO* back onto the C57BL/6 lineage for five generations. To circumvent any potential problems of fertility due to the lack of Snai2 [19] we utilized breeding pairs in which both parents were Snai2^+/-^ Snai3^-/-^ since we had observed no loss of fertility in the *Snai3KO* animals. This mating scheme was convenient for Mendelian analysis as 1 out of 4 progeny were predicted to be homozygous DKO. PCR amplification was performed on genomic DNA to detect the presence of WT and KO alleles for *Snai2* and *Snai3* (Figure 4A). Of the 129 progeny, 80 mice were *Snai2* ^*+/-*^ *Snai3* ^*-/-*^, 34 animals *Snai2* ^*+/+*^ *Snai2* ^*-/-*^ and 15 mice of the DKO phenotype, *Snai2* ^*-/-*^ *Snai3* ^*-/-*^ (Figure 4B). Based upon normal Mendelian ratios we would have expected about 32 DKO animals from this breeding scheme, half of which should have been female. The sex ratio of the DKO animals also suggested a bias for male animals in that of the 15 DKO mice, 14 were males and only 1 was female (Figure 4C,D). No such sex distinctions in progeny from the *Snai2* deficient lines were previously noted nor had we observed such a gender polarization in the *Snai3KO* progeny. Neither *Snai2* (chromosome 16) nor *Snai3* (chromosome 8) are on sex chromosomes.

To confirm that the DKO animals were truly deficient in the Snai2 and Snai3 proteins, testes were harvested from the DKO animals and analyzed for *Snai2* and *Snai3* transcripts (Supplemental Figure S6A) and Snai2 and Snai3 proteins (Figure S6B in File S1) (The expression of Snai1 by transcript and protein production was not altered in the testes of the DKO animals: Figure S6C,D in File S1). The DKO animals clearly lack these two Snail family gene products.

**Figure 5:**
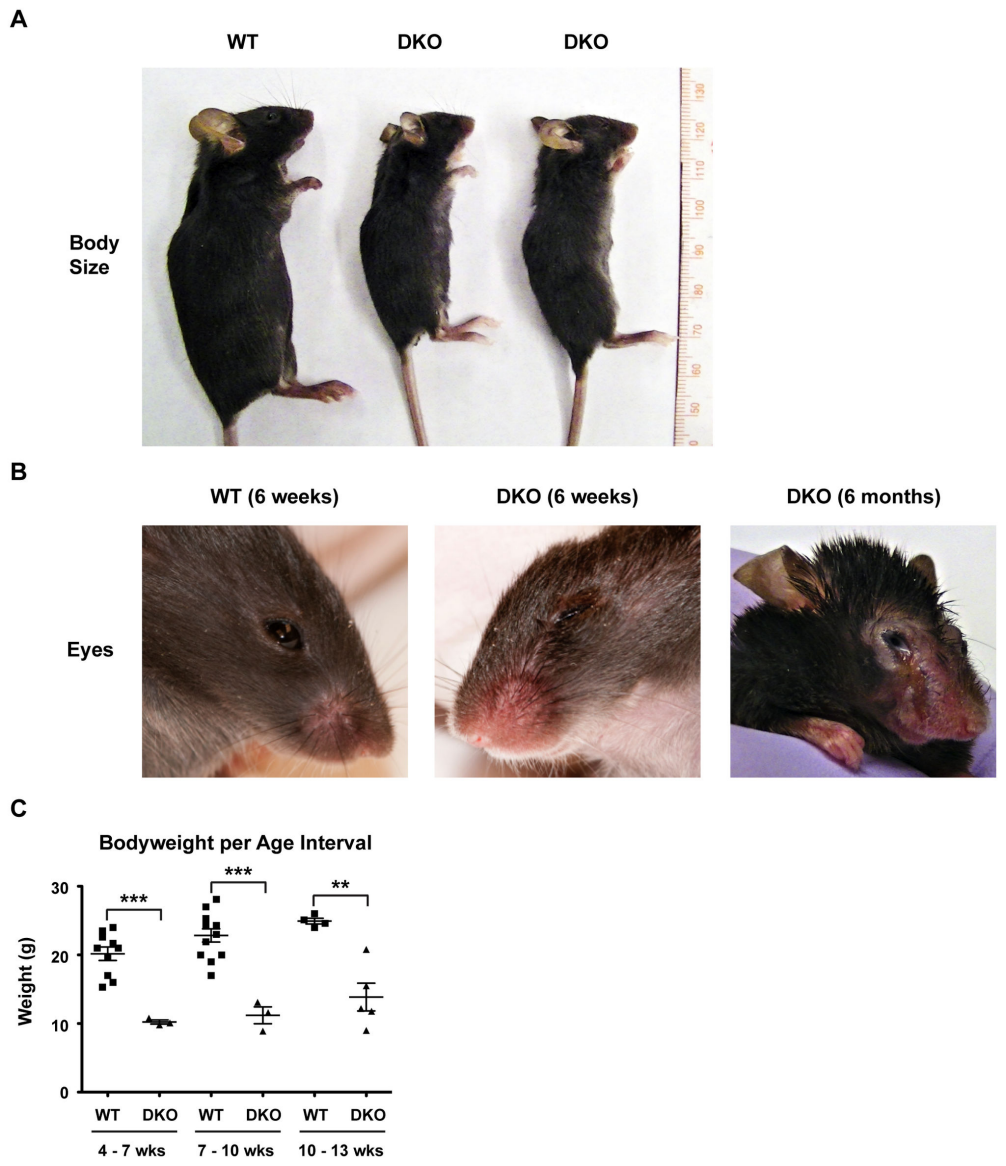
*Snai2* and *Snai3* DKO mice are developmentally stunted with ocular deformities. (A) Representative photo of age and sex matched (males) WT and DKO mice. Animals are approximately 6 weeks of age and presented alongside a ruler for reference. (B) Representative photo of WT and DKO male facial ocular area. (C) Graphical representation comparing ages (weeks, wks) and weights (grams, g) of WT and DKO animals over 3 week intervals. One-way ANOVA with Bonferroni post hoc test: ** p < 0.01, *** p < 0.001.

The physical characterization of DKO mice revealed a stunted growth phenotype. When compared with age and sex matched WT mice, DKO animals were approximately half size (Figure 5A). These animals were in general sickly, rarely survived longer than 15 weeks in the animal facility and when provided WT female mice, never generated offspring (unlike male *Snai2KO* animals which did). The DKO animals also demonstrated the eyelid anomalies previously seen in the *Snai2KO* animals [31] which was exacerbated into the loss of the eyes, presumably due to infections, in the single DKO animal that we could maintain (alone) for 6 months of age (Figure 5B). Previously the *Snai2KO* animal (on an outbred background) demonstrated a slow growth rate for the first three weeks but in later weeks grew at the same rate as WT [31]. While the *Snai2* ^*+/-*^ *Snai3* ^*-/-*^ and WT animals displayed similar body mass, Snai2^-/-^ Snai3^+/-^ animals displayed a phenotype corresponding to that previously published for the *Snai2KO* (DNS). However, the difference in body mass between WT and the DKO was obvious in animals maintained for 13 weeks (Figure 5C) and longer (the 6 month DKO shown in Figure 5B), and much more dramatic than that previously reported for the *Snai2* deficient line.

**Figure 6:**
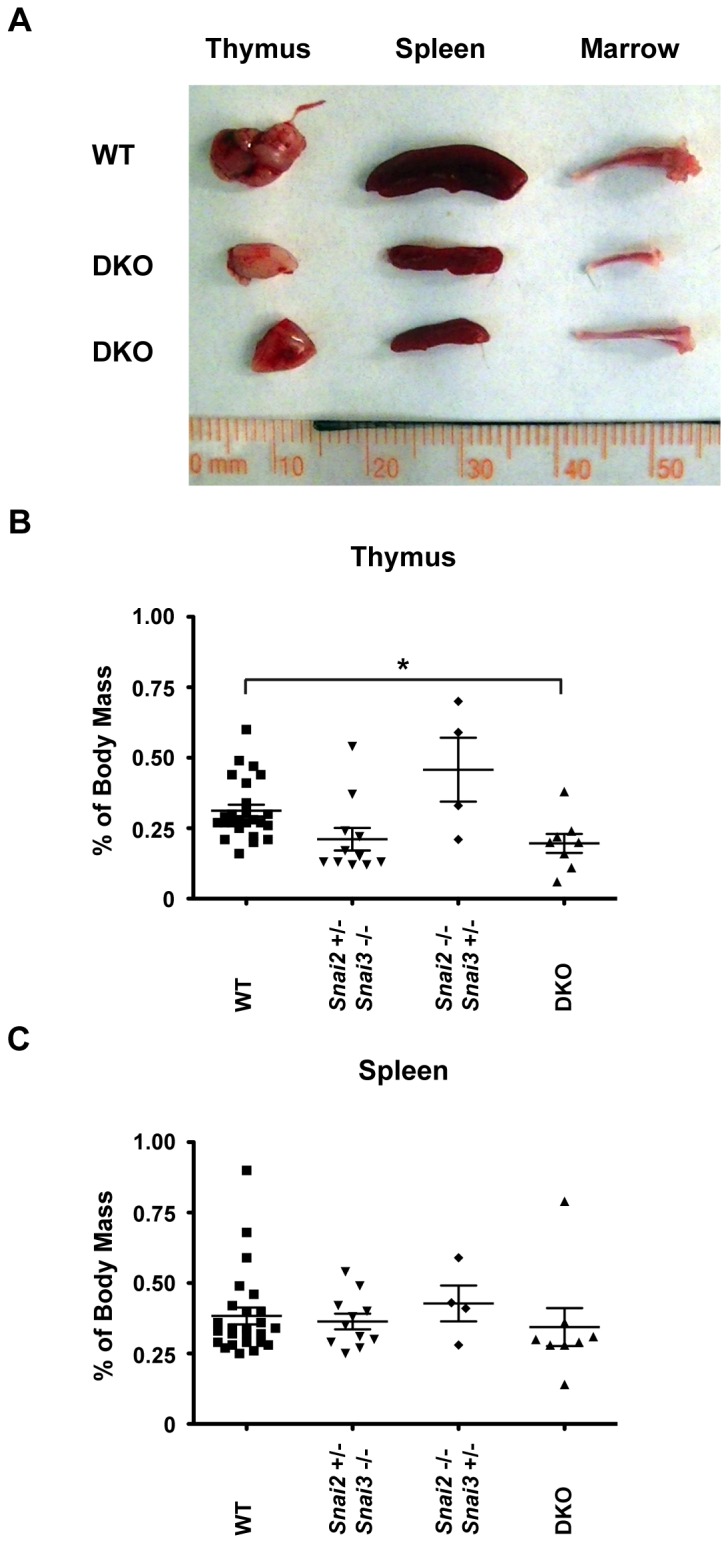
*Snai2* and *Snai3* DKO lymphoid organs are reduced in size but present a healthy appearance. (A) Representative photo of thymus, spleen, and bone marrow dissected from age and sex matched WT and DKO animals. Organs are presented with a ruler for reference. (B–C) Quantification of thymic (B) and splenic (C) mass among different iterations of *Snai2* and *Snai3* knockouts. % of Body Mass = (Organ Weight (mg) / Body Weight (mg)) X 100, One-way ANOVA with Bonferroni post hoc test: * p < 0.05.

Next we examined various lymphoid organs for gross morphology. The thymus, spleen and bone structure from DKO mice had a normal outward appearance (Figure 6A) although the sizes of the lymphoid organs were smaller in the DKO animal. When normalized to body weight, the DKO thymus was significantly smaller compared to WT (Figure 6B). No significant changes were seen in splenic mass between WT and DKO animals (Figure 6C). Additionally, Snai2^+/-^ Snai3^-/-^ and Snai2^-/-^ Snai3^+/-^ mice had organ masses comparable to WT mice.

**Figure 7:**
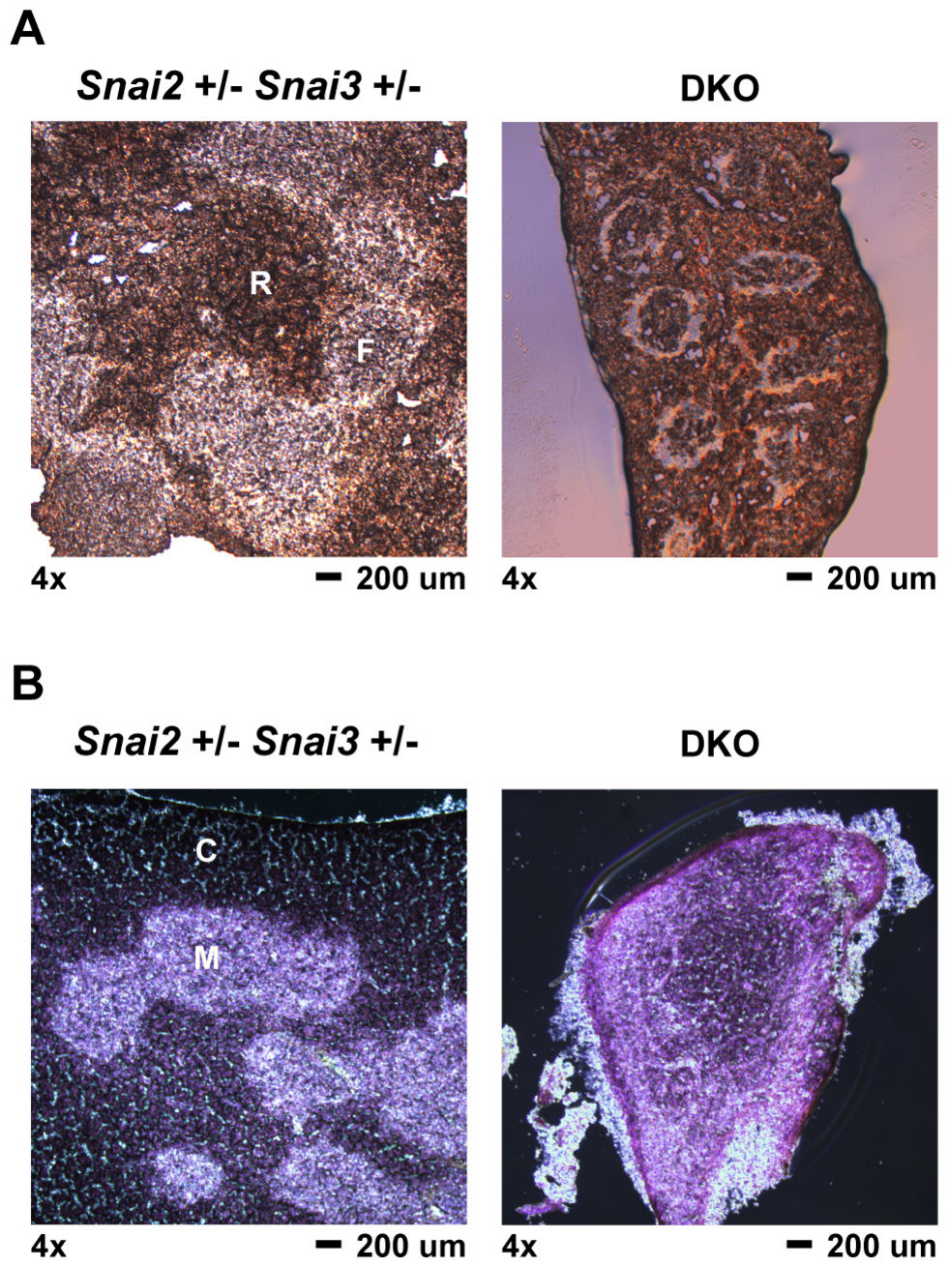
Histological analysis of 4 week old DKO spleen and thymus. Spleen and thymus were dissected from 4 week old Snai2^+/-^ Snai3^+/-^ and DKO animals. Organs were processed for histological analysis as described in the *Materials*. Tissue sections were cut to an approximate thickness of 10 µm. Representative images are shown for all genotypes and tissues assayed. (A) Splenic sections were left unstained and viewed by brightfield microscopy. 4x magnification of spleen sections from the Snai2^+/-^ Snai3^+/-^ and DKO. F = lymphoid follicle; R = red pulp. The DKO spleen is reduced in size compared to the Snai2^+/-^ Snai3^+/-^ spleen. (B) Thymus sections were stained with hematoxylin and eosin to differentiate between thymic cortex (darker stain in Snai2^+/-^ Snai3^+/-^) and thymic medulla (lighter stain in Snai2^+/-^ Snai3^+/-^): Snai2^+/-^ Snai3^+/-^ (left panel) and DKO (right panel). C = cortex; M = medulla. The DKO thymus is reduced in size compared to the Snai2^+/-^ Snai3^+/-^ thymus.

Histological analysis of the 4wk DKO animal’s spleen (Figure 7A) demonstrated a relatively normal splenic germinal center organization even though the spleen itself was about a third the size of the age matched *Snai2* ^*+/-*^ *Snai3* ^*+/-*^ spleen. A previous report showing splenic cross sections of *Snai2* deficient animals had similarly shown normal follicle structure [19]. The follicles in the DKO were ringed with a lighter, cell deficient zone that was not evident in the *Snai2* ^*+/-*^ *Snai3* ^*+/-*^ spleen. The thymus of the 4wk DKO animal demonstrated an unusual morphology with a decreased quantity of the densely staining, lymphocyte rich cortex region compared to the lighter staining medulla region (Figure 7B). This is in contrast to the age matched *Snai2* ^*+/-*^ *Snai3* ^*+/-*^ thymus sample which demonstrated normal morphology.

**Figure 8:**
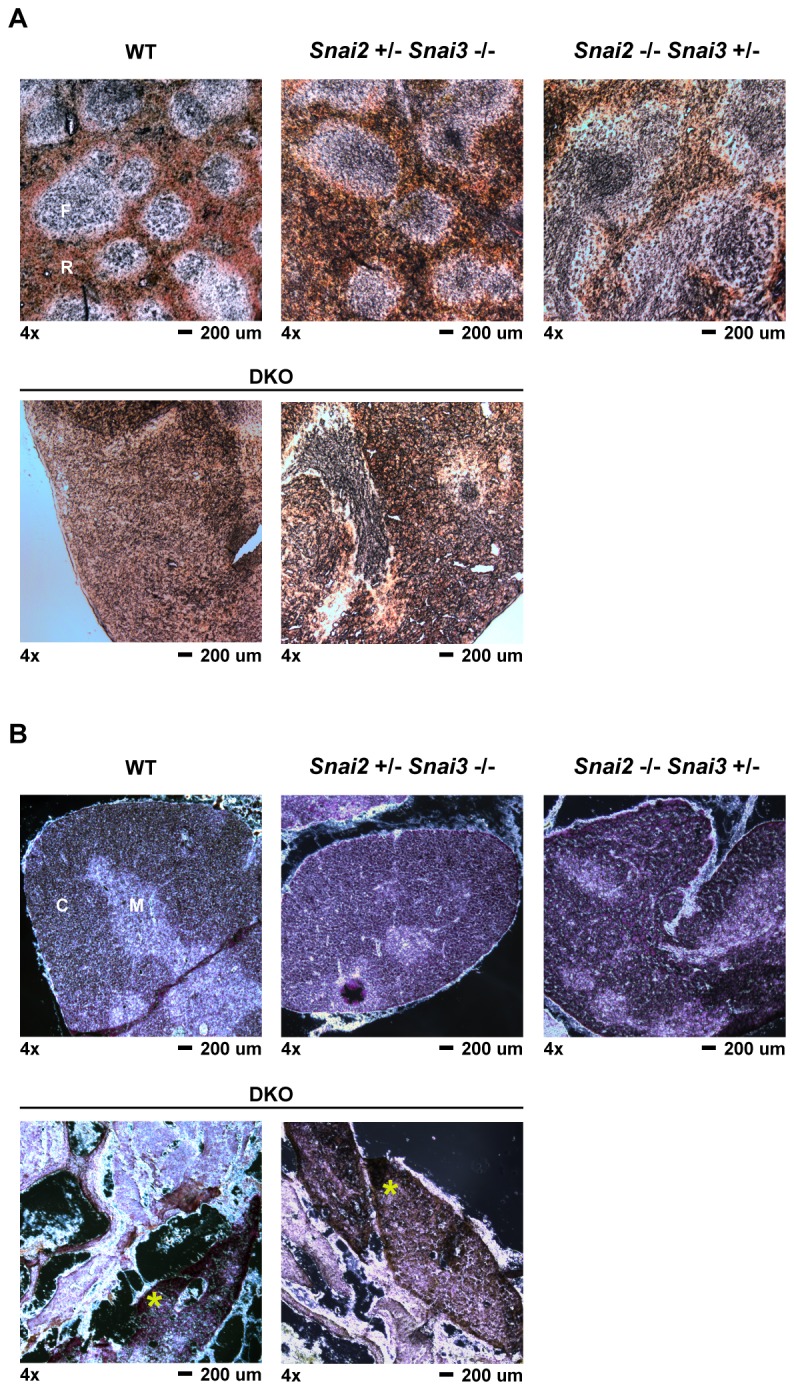
Histological analysis of six month DKO spleen and thymus tissues. Spleen and thymus tissues were harvested from six month old mice and processed as described in the *Materials*. Tissue sections were cut to an approximate thickness of 10 µm. All images were captured at 4x magnification. Representative images are shown for all genotypes and tissues assayed with two different sections shown for the DKO samples. (A) Spleen samples from the animals as marked. Sections were kept unstained and viewed via light microscopy for easier assessment of follicular versus red pulp areas of the spleen. F = lymphoid follicle; R = red pulp (B) Thymus sections were stained with hematoxylin and eosin to differentiate between thymic cortex (darker stain) and thymic medulla (lighter stain). C = cortex; M = medulla. An * is shown in the DKO sample to highlight the localization of thymus-like epithelial tissue.

Histological analysis of the 6 month old DKO spleen and thymus (Figure 8) continued to show alterations in structure. As shown in Figure 8A, the spleens of the WT, *Snai2* ^*+/-*^ *Snai3* ^*-/-*^, *Snai2* ^*-/-*^ *Snai3* ^*+/-*^ and DKO animals all possessed germinal centers, however, there was a progressive lack of organization of the follicles in the compound heterozygotes culminating in the DKO which demonstrated the greatest degree of follicle disorganization. The DKO spleens had fewer follicles interspersed in a dense staining cellular matrix. Analysis of the thymus from the DKO and compound heterozygote animals also demonstrated an altered medulla and cortex organization (compared to WT) (Figure 8B). The DKO thymus morphology was the most dramatic in which the thymus was largely involuted and fibrotic. A small section of relatively normal thymus tissue in the DKO sample is marked in the figure (yellow asterisk) and appears to contain both medulla and cortex like thymic cellular populations.

### Animals lacking *Snai2* and *Snai3* demonstrate more profound immune cell deficiencies than singe KO animals

**Figure 9:**
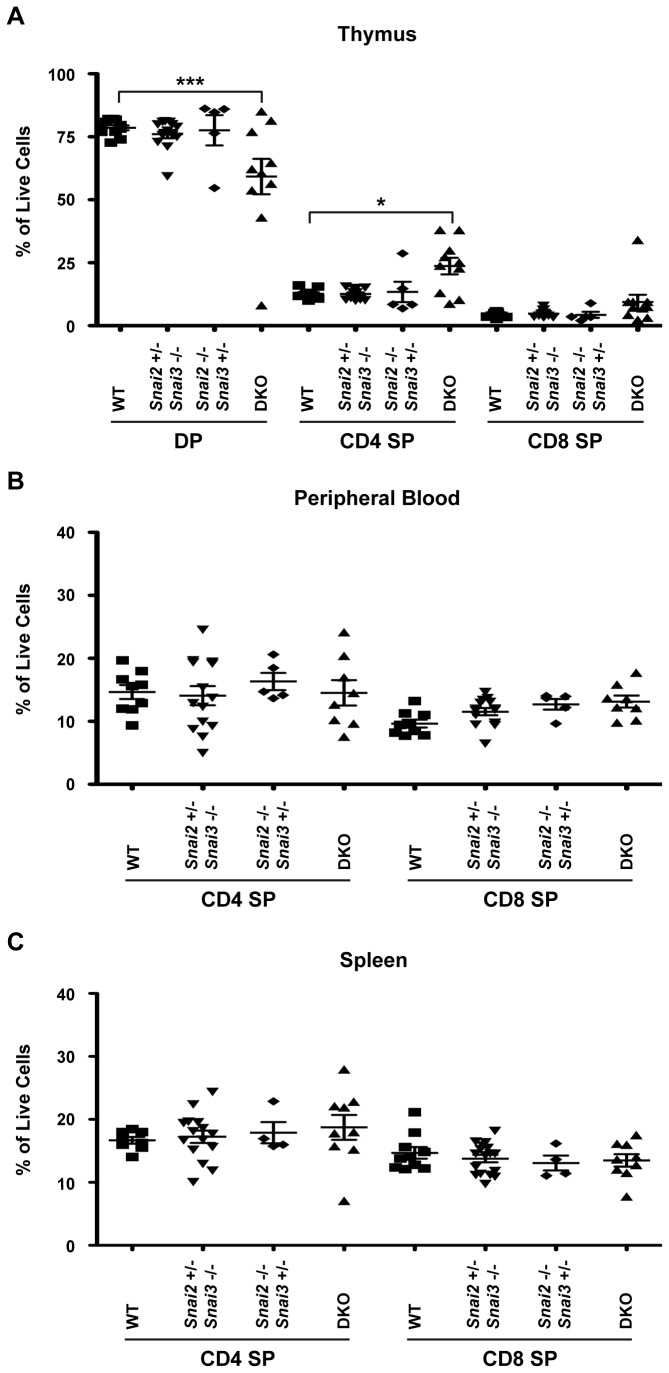
Double positive thymocytes are reduced in favor of an increased CD4^+^ single positive population in the *Snai2* and *Snai3* DKO. FACS analysis was performed to assess T cell populations in the thymus (A), peripheral blood (B), and spleen (C). Cells were assayed for CD4 and CD8 cell surface staining. DP = CD4^+^CD8^+^ double positive cells, CD4 = CD4^+^ single positive, CD8 = CD8^+^ single positive, Results are presented as a percentage of total cells analyzed. One-way ANOVA with Bonferroni post hoc test: * p < 0.05, *** p < 0.001.

DKO animals (along with controls) were analyzed for T, B and myeloid cells in the appropriate anatomic compartments. Previously the *Snai2KO* animals had been described as possessing normal numbers of B and T cells along with normal circulating blood cell counts compared to WT [13], and, as noted above, we did not observe any alterations in such cell populations in the *Snai3KO* animal. We first analyzed the DKO animals for T cell populations. FACS analysis of a single DKO animal (compared to a WT animal) for T cells in the thymus, peripheral blood and spleen is shown in Figure S7 in File S1. These analyses were expanded with additional animals possessing more diverse genotypes for quantification of cell lineages (Figure 9). These analyses revealed a significant reduction of thymic CD4^+^CD8^+^ DP cells in the DKO along with a significant increase in CD4^+^ SP cells (Figure 9A). The observed trend for increased CD8^+^ T cells did not rise to the level of statistical significance. Interestingly, there was no difference in thymic T cell populations between WT mice and mice lacking the Snai2 protein, contrary to the previous analysis of *Snai2KO* animals on a mixed genetic background [19]. Further analysis of T cells in the peripheral blood (Figure 9B) and spleen (Figure 9C) of the DKO demonstrated normal distributions of both CD4^+^ and CD8^+^ SP cells. These data suggest that any alterations in T cell phenotypes in the thymus of the DKO animal are not perpetuated in the periphery with mature CD4^+^ and CD8^+^ T cells.

**Figure 10:**
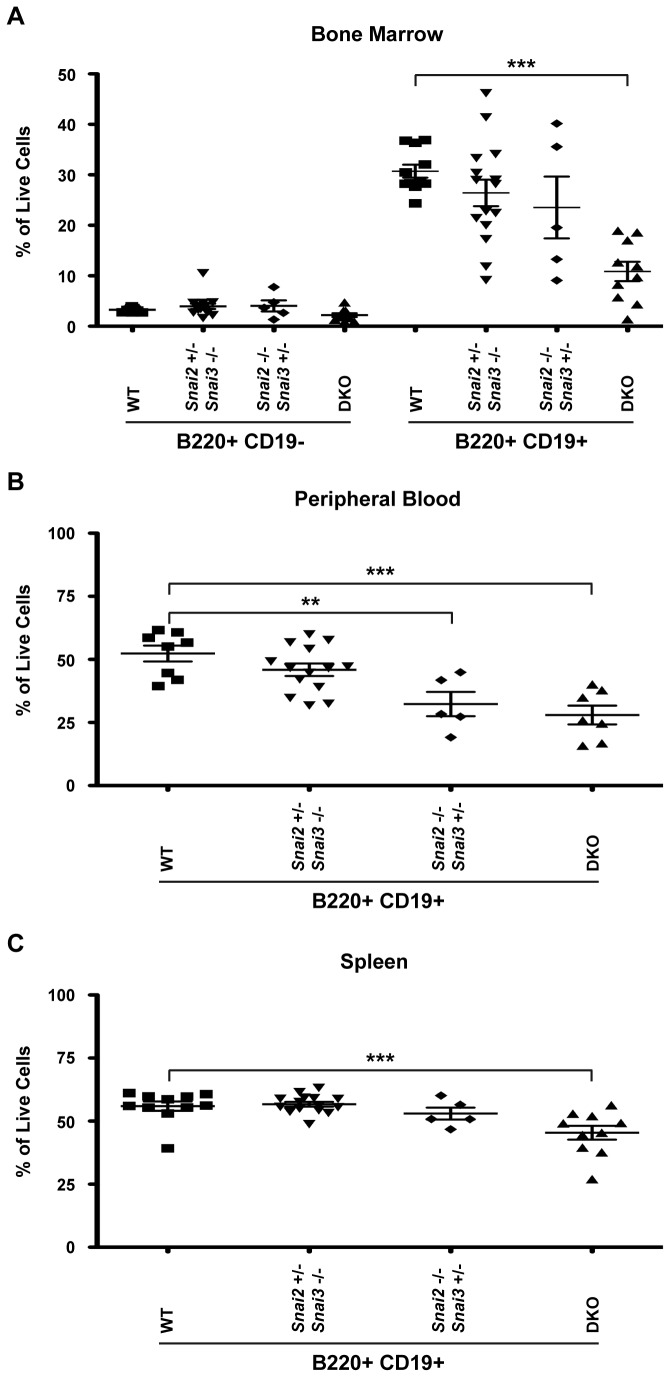
*Snai2* and *Snai3* DKO mice demonstrate a severe impairment in B cell development. FACS analysis was performed to assess B cell populations in the bone marrow (A), peripheral blood (B), and spleen (C). B cell populations were assessed using surface staining for B220 and CD19. In the bone marrow (A), B220^+^CD19^-^ (pre-pro-) and B220^+^CD19^+^ (pro-, pre-, immature, and mature re-circulating) cells were assayed. In the peripheral blood (B) and spleen (C), B cells were defined as B220^+^CD19^+^. Results are presented as a percentage of total cells analyzed. One-way ANOVA with Bonferroni post hoc test: ** p < 0.01, *** p < 0.001.

Similar protocols were employed to analyze B cell constituents in the bone marrow, peripheral blood and spleen of the DKO animals. A representative FACS analysis of a WT and DKO animal is shown in Figure S8 in File S1 using the co-expression of B220 and CD19 to quantify immature and mature B cells, and B220^+^CD19^-^ staining in the marrow for the identification of immature but committed B cell precursors. These analyses are quantified in Figure 10. The DKO animals possess fewer B220^+^CD19^-^ and significantly less B220^+^CD19^+^ bone marrow B cells than the WT controls while the heterozygous controls show intermediate levels of B220 ^+^CD19^+^ B cells in the marrow (Figure 10A). Reduced percentages of B cells in the DKO are maintained in the blood as is the intermediate phenotype of the heterozygote animals (Figure 10B). Of these, the *Snai2* ^*-/-*^ *Snai3* ^*+/-*^ animals show a greater B cell deficiency than the *Snai2* ^*+/-*^ *Snai3* ^*-/-*^ animals. In the spleen, the percentages of B cells are only significantly reduced in the DKO animal compared to WT or the heterozygote animals (Figure 10C).

**Figure 11:**
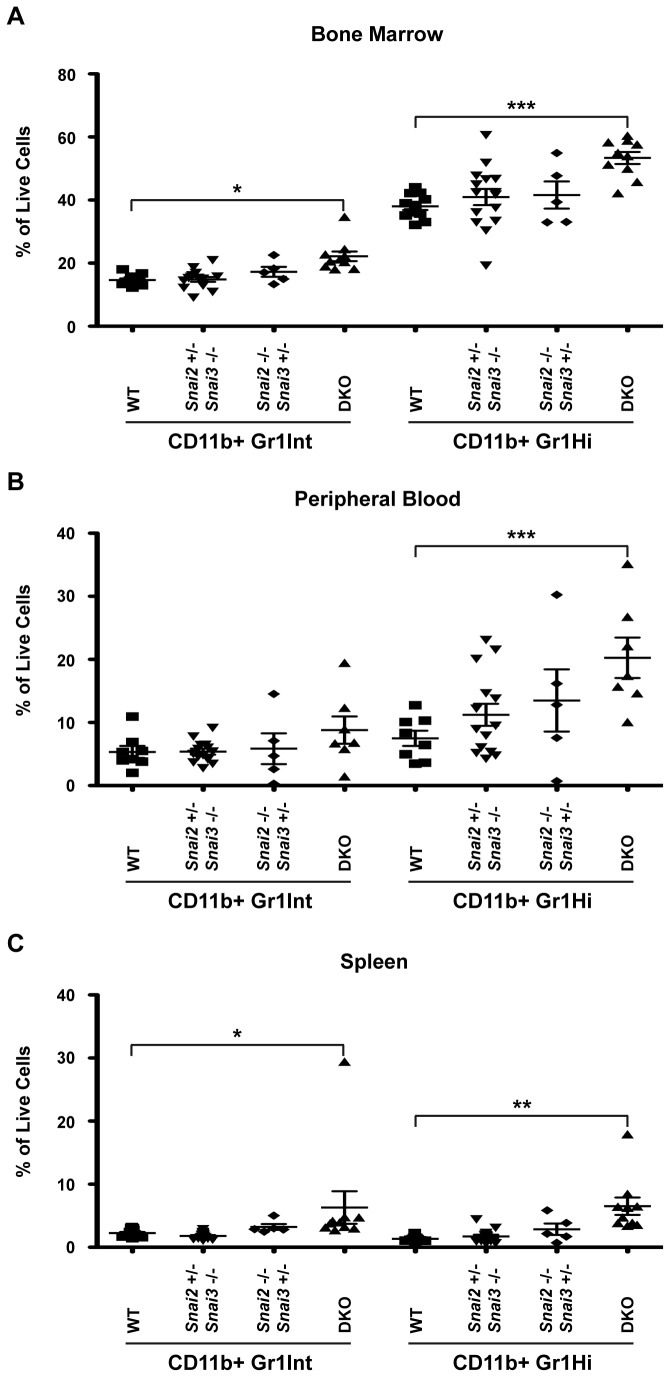
Myeloid populations are enhanced in the *Snai2* and *Snai3* DKO. FACS analysis was performed to assess myeloid cell populations in the bone marrow (A), peripheral blood (B), and spleen (C). Cells were stained for CD11b and Gr1. Macrophages are identified by CD11b^+^ Gr1^Int^ staining. Neutrophils (representative of granulocytes) are distinguished by CD11b^+^ Gr1^Hi^ staining. Results are presented as a percentage of total cells analyzed. One-way ANOVA with Bonferroni post hoc test: * p < 0.05, ** p < 0.01, *** p < 0.001.

The other major cell lineage that develops in the marrow is the myeloid lineage that gives rise to macrophages (defined as CD11b^+^Gr1^Int^), neutrophils (CD11b^+^Gr1^Hi^) and other cells types. Representative FACS plots for WT and DKO mice are provided in Figure S9 in File S1, and the quantitative analysis of these data, along with heterozygotes, is shown in Figure 11. In the bone marrow, the apparent loss of maturing B cells resulted in significant increases in both macrophage and neutrophil populations (Figure 11A). When peripheral blood was examined, PMN were significantly enhanced in the DKO (Figure 11B). WT animals analyzed ranged from ~2–10% circulating neutrophils, however, the DKO animals averaged ~20% neutrophils in the blood. While infections can cause the increased mobilization of neutrophils into the bloodstream, no infections were noted in the DKO animals at the time of analysis suggesting the elevated levels of neutrophils in the bloodstream were due to enhanced production of this cell type. Macrophages in the blood were also enhanced albeit not to a significant level. This may in part be a reflection of the propensity of macrophages to migrate and establish tissue residence. In the spleen, macrophages and neutrophils showed a significant enrichment in the DKO (Figure 11C).

**Figure 12:**
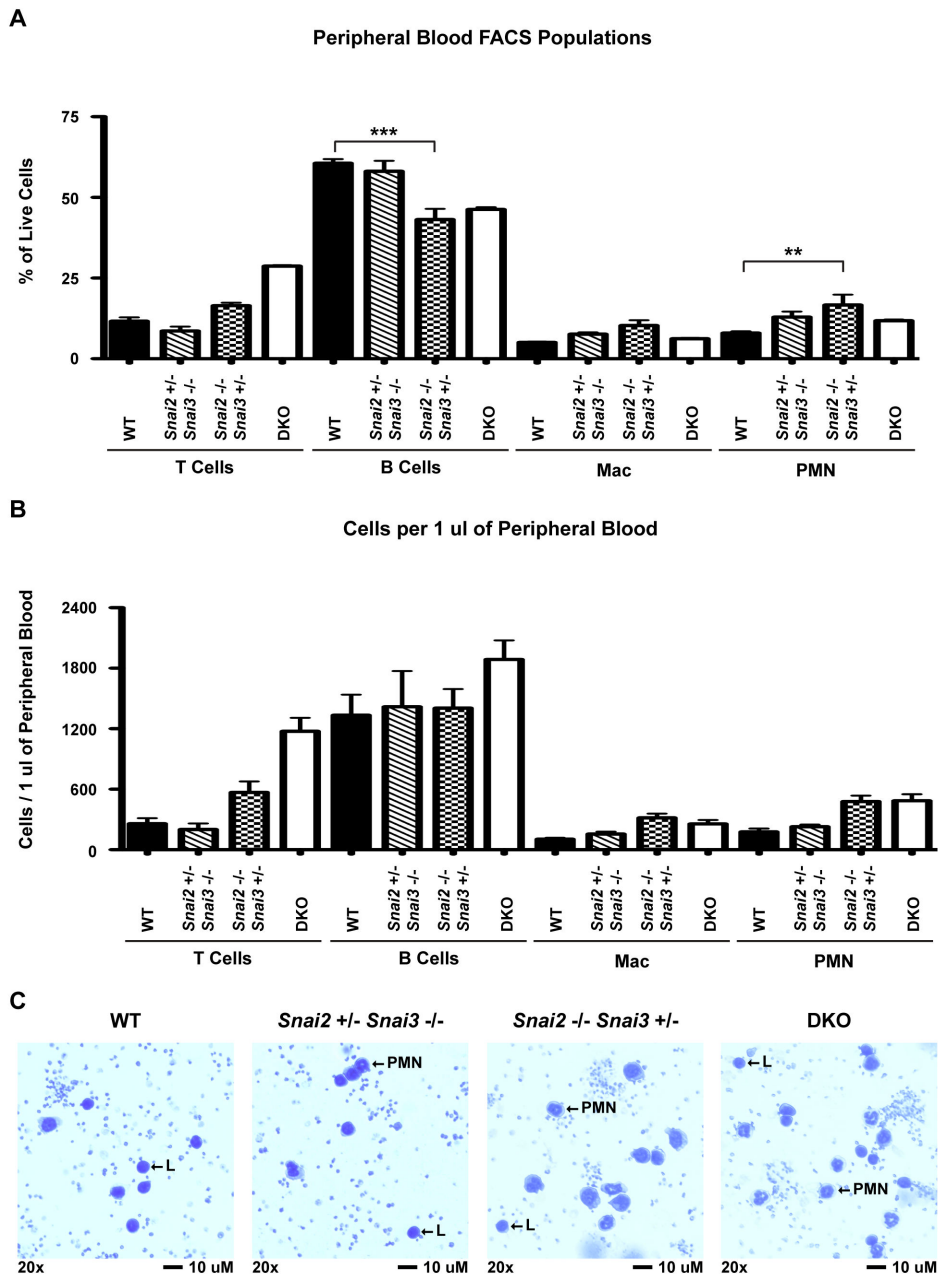
Circulating hematopoietic profile of six month old mice. Retro-orbital bleeds were performed three times for each animal assayed. Three mice were analyzed for WT, Snai2^+/-^ Snai3^-/-^, and Snai2^-/-^ Snai3^+/-^ genotypes. Only one DKO animal survived to six months of age. FACS was used to assess overall percentages (A) and absolute numbers of each lineage (B) within the peripheral blood. Significance was tested using one-way ANOVA followed by the Bonferroni post hoc test. ** p < 0.01, *** p < 0.001 (C) Snai2^-/-^ Snai3^+/-^ and DKO animals display increased lymphocytes and neutrophils in circulating blood. Cytospins were performed with 30 µl of peripheral blood. Slides were stained with Wright-Giemsa to differentiate between blood cell types. 20x images were photographed and representatives for each genotype are shown.

The analysis of cell types in the blood of the DKO animals was further investigated using older (6 months) WT and compound heterozygote animals and the single DKO animal that survived to 6 months. Blood samples from such mice were analyzed for both percentages of cell types (Figure 12A), total numbers of cells per 1µl of blood (Figure 12B) and analyzed by cytospin (Figure 12C). The percentages of blood cells in these animals showed a skewing towards greater percentage of T cells and PMN in the animals lacking both *Snai2* alleles. Total numbers of cells in the blood of the Snai2^-/-^ Snai3^+/-^ animals were also increased, compared to WT and the Snai2^+/-^ Snai3^-/-^ mice via the expansion of PMN, macrophage and T cell numbers. The elevated numbers of cells in the blood of the DKO and Snai2^-/-^ Snai3^+/-^ mice were also evident in the cytospins in which greater numbers of myeloid and lymphoid cells were visible when compared to the WT or Snai2^+/-^ Snai3^-/-^ animals. 

## Discussion

In this study we have examined the roles of the transcriptional repressor proteins Snai2 and Snai3 on the development of the mouse and immune cell lineages. The high level of expression of *Snai3* in skeletal and cardiac muscle tissue and thymus suggested that deletion of the gene might be an embryonic lethality and lead to T cell development anomalies. Instead we found that deletion of *Snai3*, either only in T cell lineages via *Lck-Cre* or in the entire animal had no effect upon viability, reproduction, physical appearance or immune cell derivation. Therefore, unlike *Snai1*, *Snai3* is not an essential gene.

The absence of phenotype associated with the lack of the Snai3 protein either suggested that the protein has only minimal function or that another member of the family functionally complements its absence. While *Snai1* expression is demonstrable in many bone marrow lineages (Figure 2C) animals lacking the factor do not survive. Alternatively *Snai2* expression is confined to the more immature cell types of the marrow and thymus (Figure 2D); a previous report documented the expression of *Snai2* in early lymphoid and myeloid precursors as well as hematopoietic stem cells [13]. *Snai2* deficient animals are viable but do demonstrate a number of deficiencies. *Snai2* deficient animals were described as possessing swollen eyelids that enhanced infections of the eye and displayed moderate postnatal growth retardation that was most evident in animals prior to weaning [31]. The numbers of T and B cells and their mitogenic responses were not altered in the *Snai2* deficient animals nor were there differences in blood counts between WT and *Snai2* deficient animals [13]. There were, however, increased numbers of hematopoietic colony forming units in the marrow and spleen of such animals [13,20]. A previous analysis of *Snai2* expression in the thymus and spleen using a Snai2-LacZ fusion protein had identified only a small subset of scattered cells of unknown identity expressing the protein: no report of marrow cell expression of the Snai2-LacZ fusion protein was noted [37]. Although *Snai2* deficient animals were described as having normal T and B cell numbers [13], a different report did document the skewing of immature T cell populations in the thymus of a *Snai2* deficient animal, the same populations that we observed high levels of *Snai3* expression [19]. Therefore we chose to create a double knockout animal of both Snai2 and Snai3 proteins to determine if T cell populations (and those of other bone marrow lineage cells) had enhanced developmental anomalies compared to those found for the *Snai2* deficiency alone.

The *Snai2/Snai3* deficient, DKO animals demonstrated a number of phenotypes enhanced over the single KO lines. At the most superficial layer, the DKO animals were dramatically reduced in size at birth compared to WT and maintained the runted phenotype for the life of the animal (Figure 5). This size differential was much greater than that previously described for the *Snai2KO* animals. There was also a sex specific skewing of the DKO progeny (Figure 4). While males were generated at near Mendelian levels, the number of female progeny was well below expectations. Indeed of the 15 DKO animals analyzed for this study only one was female. A similar gender skewing was not reported for the *Snai2* deficient animals and we did not observe this with the *Snai3KO* line. Most instances of sex specific lethality map to the X-chromosome yet neither *Snai2* or *Snai3* are on that chromosome. The *Snai2/3* gene products, however, may influence the expression of other genes on the sex chromosomes. For example, deletion of components essential in initiating and maintaining X-chromosome inactivation (XCI) consistently results in a female-specific block in embryonic development [38,39] and their expression could be modulated by Snai2/Snai3. Long non-coding RNAs (lncRNAs) such as *Xist* function to initiate XCI. In contrast, additional effectors such as *Tsix* act to directly inhibit the function of *Xist* [40]. Thus Snai2/Snai3 may modulate the expression of these components via the recruitment of HDACs. An alternative explanation for the sex skewing of the DKO offspring may be by complementation via Snai1 in male offspring that is not readily available to females *in utero*. All three Snail family members are expressed in the testes (Figure 3) such that altered expression of *Snai1* in the sperm of the DKO animals may negatively influence the ability of the XX fertilized egg to survive. We have not identified at what point in gestation female DKO animals are lost.

The analysis of the hematopoietic system of the DKO indicated that gene dosage of Snail family members can influence the development of bone marrow derived cells and the structure and organization of lymphoid tissues. For example, the thymus architecture of the DKO is different than WT or the compound heterozygotes (Figures 7, 8). Maturing T cells lacking both Snai2 and Snai3 proteins showed a lower CD4^+^CD8^+^ DP population than either of the compound heterozygotes or WT as well as an elevated CD4^+^ population (Figure 9). CD8^+^ cells demonstrate much higher expression of *Snai3* than CD4^+^ cells (Figure 2) thus the lack of the two transcriptional repressors Snai2 and Snai3 in CD8^+^ cells may promote the development of the CD4^+^ lineage. If this is the case, then negative and positive selection of such cells may have been compromised in the thymus leaving such animals vulnerable to autoimmune traits that would be evident in older mice [41–43]. Unfortunately the severe running of the DKO animals precludes such long-term analyses but they can be addressed in the future using T cell specific deletion of the Snai2/Snai3 proteins. A previous report [20] detailed the role of Snai2 in controlling the generation of the stem cell precursors that feed into the T, B and myeloid lineages analyzed in this paper. That report did not describe deficiencies in such precursors and actually demonstrated an enhanced ability of stem cell expansion following insult in the *absence* of Snai2. Therefore it is unlikely that the alterations in T, B and myeloid precursors observed in this report of the DKO animals is due to compromised stem cell development, and is instead due to alterations in gene expression in more committed lineages.

Similar to the analysis of the thymic precursors, the development of the B cell and myeloid lineages in the bone marrow suggests that the Snail family of proteins is important for the early development of these lineages. Examination of B cell populations in the DKO animals showed a striking reduction that was initiated in the bone marrow (Figure 10A). Percentages of the earliest forms of committed B cells (B220^+^CD19^-^: pre-pro-) were reduced by approximately 30 percent. This difference was further amplified in the more mature bone marrow subsets (B220^+^CD19^+^) (~65% reduction) suggesting an initial deficiency in cells committing to the B cell lineage in the DKO animals. The loss of only Snai2 also negatively impacts the development of marrow B cells and peripheral blood levels of the cells however, only the DKO animal shows significant loss of B cells in the splenic populations again suggesting that the combined loss of both Snai2 and Snai3 in these cell lineages has a greater developmental impact than the loss of only a single family member. The loss of B cells in the DKO may be related to the anti-apoptotic responses associated with the Snail proteins [13,18,44,45]. V(D)J recombination of B (and T) cells primes lymphocytes to undergo apoptosis in the event that the recombinations are non-productive [46–48]. The DKO animals may have heightened sensitivity to such apoptotic signals reducing the production of marrow and bloodstream B cells yet allowing for the eventual colonization of the peripheral lymphatic organs such as the spleen. Indeed the survival of the DKO B cells in the spleen may be enhanced by the actions of BAFF that leads to the expression of anti-apoptotic control proteins [49,50].

The enhanced development of myeloid cells in the DKO animal may be due directly to the absence of the Snai2/Snai3 proteins, or indirectly, to the relative absence of the B cell precursors. Future analysis of progenitor populations such as the granulocyte-monocyte (GMP), common myeloid (CMP) and common lymphoid (CLP) progenitors may begin to shed light onto this question.

The data presented in this manuscript offer a glimpse into the possible functions of the Snail family of proteins in bone marrow cell derivation. Previously we had shown that over-expression of *Snai3* in the earliest of stem cell precursors (via stable *Snai3*-encoding retrovirus infection of stem cells in bone marrow chimera animals) blocked lymphocyte development and shunted bone marrow development into the myeloid lineage [21]. Since Snai3 binds to the canonical E-box motif with an internal GC rich di-nucleotide, the over-expression of the protein would have blocked the binding of other E-box proteins that recognize such sites. The Id proteins are helix-loop-helix proteins that dimerize with E-box binding proteins and block their transcriptional activation potential [51,52]. Ectopic expression of Id in a bone marrow chimera model also blocked the development of lymphocytes, instead shunting cells into the myeloid lineages [53]. These data suggest that expression of an undefined E-box protein is critical for the derivation of the lymphoid lineage: inhibition of this E-box protein (or multiple proteins) by the over-expression of Id or Snai3 thus blocks its function and blocks lymphocyte development. The identification of such an E-box transcriptional activator and the genes it targets would enhance our understanding of myeloid versus lymphoid development. We would predict that such target genes would possess GC-rich di-nucleotide E-box sites.

The removal of Snai2 and Snai3 in the DKO, however, demonstrates their functions in hematopoiesis at stages past the lymphoid versus myeloid differentiation decision. The stages of T and B cell development are altered and perhaps retarded from their normal time line of differentiation, yet mature, end stage T and B cells are still obtained in the DKO animals. The E2A gene encodes two proteins, E12 and E47, that bind to GC-rich di-nucleotide E-boxes (the same sequences favored by the Snail family of proteins). E12 and E47 proteins are critical for the early stages of B cell and T cell development [54,55]. Animals lacking E2A gene products show enhanced CD4^+^ SP cells in the thymus and decreased CD4^+^CD8^+^ DP cells, similar to our observations with the *Snai2/Snai3* DKO animals. In animals lacking the E2A proteins, the impact on B cell development is dramatic with very few splenic B cells, and marrow B cells blocked at the first stages of immunoglobulin rearrangement. Interestingly the over-expression of E47 leads to the enhanced expression of *Snai3* [54] (and Snail family and E47 proteins directly interact [5]) suggesting that the expression and functions of the E2 and Snail family products are coordinated in the maturation of bone marrow derived cells.

In summary we have shown that the sum of eliminating both *Snai2* and *Snai3* provides for a more profound phenotype than seen with animals deficient in only *Snai2* or *Snai3*. The absence of both proteins is tolerated by male offspring, but only rarely by females, however all DKO animals are runted and fail to thrive. The development of T and B cells is not blocked, but is inhibited and may give rise to cells with impaired functions. These data suggest that the development of the mouse and of bone marrow lineages requires the actions of the Snail family of proteins to inhibit E-box activating proteins and that the loss of both of the Snai2 and Snai3 proteins can lead to phenotypes that are not evident in the animals lacking only one such protein.

## Materials and Methods

### Animal strains and care

Animals were housed in the Animal Resource Center (University of Utah Health Science Center, Salt Lake City, UT) according to the guidelines of the National Institute of Health for the care and use of laboratory animals. All animal protocols were reviewed and approved by the University of Utah Institutional Animal Use and Care Committee. C57BL/6 mice (Stock #000664) and B6.SJL-Ptprc Pepc/BoyJ (Stock #002014) were obtained from The Jackson Laboratories. B6; 129S1-Snai2^tm2Grid^/J mice (Stock #010617) were obtained from The Jackson Laboratories and backcrossed to C57BL/6 for five additional generations. Mice of at least 4 weeks of age were used for all experiments.

### Snai3 knockout construct and gene targeting

A 12kb fragment encompassing the entire *Snai3* genomic region (NCBI 30927) was screened out of an 129 lambda phage library using probes specific to *Snai3* coding sequence (NM_013914) and cloned into the NotI and XbaI sites of pSK creating pSK-*Snai3*. The XbaI site of pSK-*Snai3* was changed to NotI and the 12kb *Snai3* fragment was digested by NotI and cloned into ΔSalpSK plasmid creating ΔSalpSK-*Snai3*. LoxP oligos were ligated together and cloned into the PacI site (first intron) of ΔSalpSK-*Snai3*. The NotI site was changed to SalI in the PL451 neo cassette construct and the entire neo cassette (LoxP-Frt-neo gene-Frt) was excised by SalI digest and cloned into the SalI site of ΔSalpSK-*Snai3* creating a complete pSK-*Snai3*-KN targeting construct, see Figure 1. The entire *Snai3*-KN targeting construct was excised by NotI digest and cloned into the TK1-TK2 vector. The TK1-TK2 vector was linearized by FseI digest and used to make targeted ES cells for blastocytes injection by standard transgenic mouse techniques at the University of Utah Knockout/Transgenic Mouse Core.

### RNA preparation, cDNA synthesis and RT-PCR

Total RNA was isolated from cells using Illustra RNAspin Mini kit (GE Healthcare) according to the manufacturer’s instructions. cDNA was synthesized using SuperScript III First-Strand Synthesis System (Invitrogen) and purified with the GeneJET PCR Purification kit (Fermentas). Semi-quantitative RT-PCR was performed via incorporation of [^32^P] dCTP [56]. Amplification products were subjected to polyacrylamide sequencing gel electrophoresis. Products were visualized by exposure to X-ray film at -80° C. Quantitative RT-PCR was performed using Light Cycler (Roche Diagnostics) [57]. All transcript values shown are relative to *Actb* expression and are mean values ± standard error measurement (SEM). Primer sequences are provided in Table S1 in File S1.

### Immunoblotting (Western blot)

Total whole cell lysates (WCL) were generated from thymus, spleen, bone marrow, and testes via lysis in Radio-Immunoprecipitation Assay (RIPA) buffer following standard methods. Protease inhibitors were added to RIPA before use (Roche, 04693132001). Protein amounts equivalent to 2.5–5 million (M) cells were electrophoresed in 4-20% gradient SDS-PAGE gels (Thermo Scientific, 0025204). After transfer to PVDF membranes, blots were stained with Ponceau Red and cut into strips to allow for simultaneous probing of target antigens. After removing Ponceau staining via TBS-T washing, blots were blocked in 5% NFDM/TBS-T for 1 hour at room temperature. Blots were then probed at 4° C overnight with the following primary antibodies at the indicated dilutions: Snai1 (Santa Cruz, sc-10432, 1:250), Snai2 (Santa Cruz, sc-10436, 1:100), Snai3 (Harlan Bioproducts, P070240, 1:250) [21], and β-actin (Sigma, A2066, 1:10,000). The next day, blots were washed with TBS-T and incubated for 2 hours at room temperature with the following secondary antibodies: peroxidase-conjugated bovine anti-goat (Jackson ImmunoResearch, 805-035-180, 1:10,000) or peroxidase-conjugated goat anti-rabbit (Bio-Rad, 170-6515, 1:10,000). Blots were washed sequentially with TBS-T and then TBS to remove unbound antibody and excess Tween, respectively. Bound antibody was detected by standard ECL detection (Thermo Scientific, 34080) methodology and exposure to X-ray film.

### DNA isolation and genotyping

Approximately 5 mm tail sections were boiled in 50 mM NaOH for at least one hour. 1 M Tris was added to neutralize the NaOH. Following centrifugation to remove insoluble material, DNA was precipitated from supernatants following standard ethanol precipitation guidelines. *Snai2* and *Snai3* genotyping was performed with Fermentas *Taq* DNA Polymerase (EP0402) using 2 µl of DNA per reaction. Cycling parameters are available upon request. Primer sequences are provided in Supplemental Table 1. Products were electrophoresed in 2% agarose gels.

### FACS analysis and cell sorting

All experiments were performed using mice ≥ 4 weeks of age. Cells were prepared for FACS analysis as previously described [49]. The following antibodies were used with the dilutions indicated: CD16/32 (BioLegend, 101302, 1:1000), CD3-FITC (eBioscience, 11-0031-82, 1:100), CD4-PerCPCy5.5 (BioLegend, 100434, 1:200), CD8-PE (eBioscience, 12-0083-83, 1:200), CD19-FITC (eBioscience, 11-0193-85, 1:500), B220-PE (BioLegend, 103208, 1:500), CD11b-PerCPCy5.5 (BioLegend, 101227, 1:500), and Gr1-PECy7 (BioLegend, 108415, 1:2300). Population analysis was performed on the FACS Canto II (BD Biosciences) and results are presented as percentages of the total live cells analyzed per tissue. Cell sorting of specific populations was performed on the Aria Cell Sorter (BD Biosciences) at the University of Utah Flow Cytometry Core.

### Tissue Preparation and Analysis

Spleen and thymus were dissected from mice and briefly rinsed in 1x PBS. Tissues were fixed overnight at 4° C in 4% paraformaldehyde following by sequential dehydration steps in 5% sucrose: 1x PBS, 15% sucrose: 1x PBS, and 30% sucrose: 1x PBS. Following dehydration, tissues were OCT embedded on dry ice. Sections were cut to a thickness of 10 µm using a cryostat.

Hematoxylin and eosin staining were performed following manufacturer protocols. Cytospins were prepared using 30 µl of blood following lysis of erythrocytes. Slide preparations were stained with Wright-Giemsa stain (Ricca, 9380-16) to differentiate cell types. Samples were imaged with an Olympus BX-51 microscope. Image processing was performed using cellSens Dimension software (Olympus).

### Statistical analysis

One-way ANOVA followed by the Bonferroni post hoc test was utilized for all statistical measurements performed. Statistical cutoffs are noted in the figure legends.

## Supporting Information

File S1 Figure S1, S2, S3, S4, S5, S6, S7, S8, S9 and Table S1. (PDF)
