# Supplementary material for: Deletion of Snai2 and Snai3 Results in Impaired Physical Development Compounded by Lymphocyte Deficiency
Source: PLoS One. 2013 Jul 16;8(7):e69216. doi: 10.1371/journal.pone.0069216 (PMC3713067; doi:10.1371/journal.pone.0069216)
Supplement: File S1 — (PDF) [file pone.0069216.s001.pdf]

Pioli, et al. Deletion of *Snai2* and *Snai3* Results in Impaired Physical Development Compounded by Lymphocyte Deficiency

Supporting Information File S1

Figures S1-S9

Table S1

Figure S1

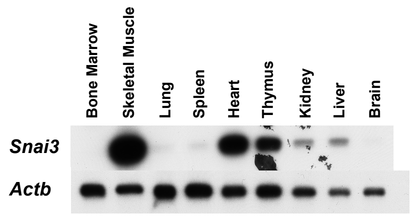

Figure S1. *Snai3* transcripts are widely expressed among mouse tissues. Total RNA was isolated from whole mouse tissues and cDNA was generated. PCR amplification was performed in the presence of [^{32}P] dCTP and products were subjected to electrophoresis in a polyacrylamide sequencing gel [47]. *Snai3* (28 cycles) and *Actb* (16 cycles) were amplified using a constant cycle number for all samples. Products were detected by exposure to X-ray film at -80°C. A representative gel image is presented from the tissues obtained from a single mouse. Primer sequences are listed in Supplemental Table 1.

Figure S2

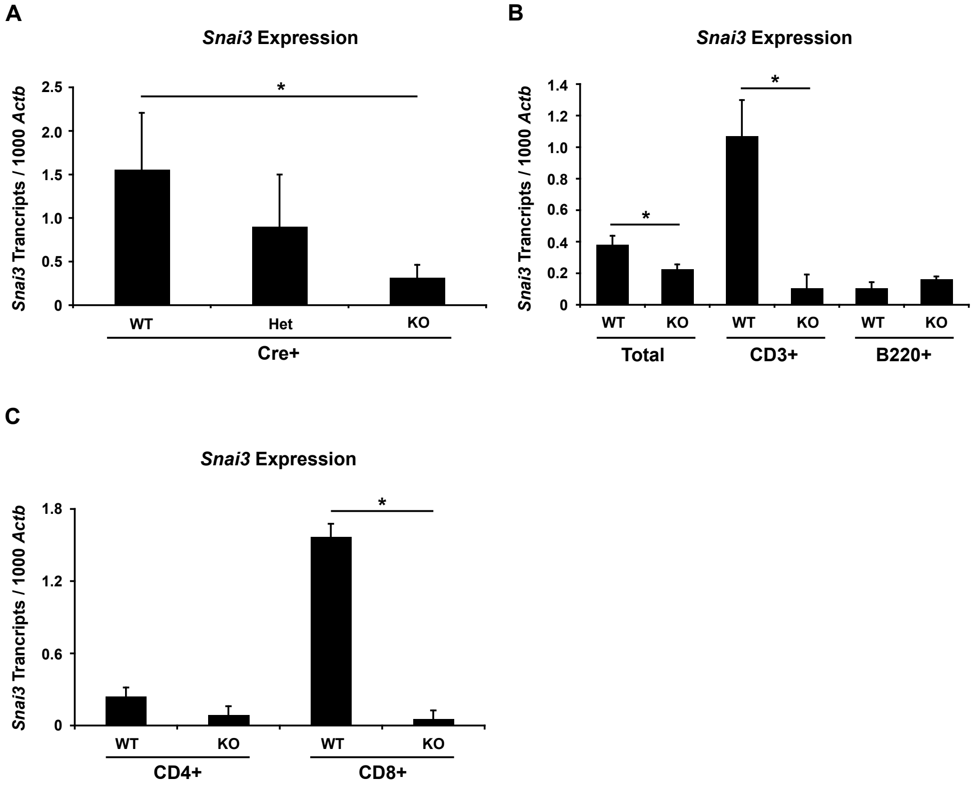

Figure S2. RT-PCR analysis of *Snai3* transcripts in *Snai3* conditional knockout mice. (A) RT-PCR analysis of *Snai3* expression in total thymus RNA prepared from *Lck-Cre*-possessing animals with WT *Snai3* genes (WT Cre^+), WT and targeted *Snai3* alleles (Het Cre^+) and both targeted *Snai3* alleles (KO Cre^+). (B) RT-PCR analysis of *Snai3* expression by cells from WT and *Snai3* targeted, Cre^+ animals (KO). Total, total splenocytes; $CD3^+$ and $B220^+$ purified subsets of T and B cells, respectively. (C) RT-PCR analysis of *Snai3* expression by $CD4^+$ and $CD8^+$ selected splenic T cells from WT and KO (both *Snai3* targeted alleles with *Lck-Cre*). Values in panels A,B,C are *Snai3* transcripts per 1000 *Actb* transcripts and are averages from n=3 mice ± standard deviation. * denotes statistically significant reduction in transcript level versus all other conditions p-value < 0.05.

Figure S3

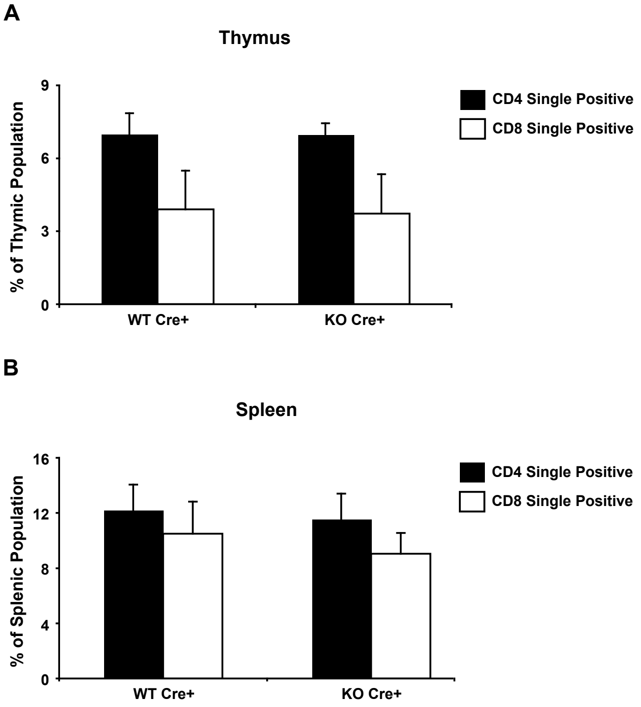

Figure S3. FACS analysis of conditional *Snai3* knockout mice. (A) FACS quantification of $CD4^+$ (black bar) and $CD8^+$ single positive cells in the thymus of WT (C57BL/6) and those with both *Snai3* targeted alleles with *Lck-Cre* (KO Cre). (B) The same single positive analysis of WT and KO T cells from the spleen. There is no significant difference in cell numbers between these two strains of mouse. Percentages are averages from n=3 mice ± standard deviation.

Figure S4

A

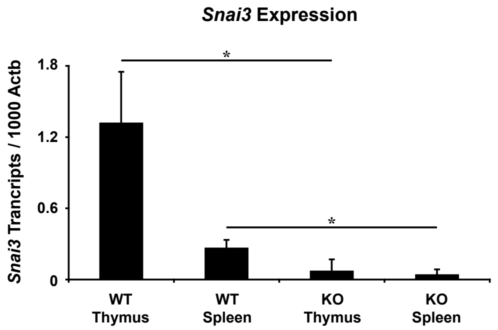

Figure S4. RT-PCR analysis of *Snai3* transcription in germline knockout mice. RNA was extracted from total thymus or total spleen tissue and analyzed for *Snai3* expression. Wild type (WT)(C57BL/6) and Full KO (germline deletion of *Snai3*) animals were used for tissue harvest. Both thymus (p-value ≤ 0.008) and spleen (p-value ≤ 0.01) had significant decreases in the *Snai3* transcript level in the KO animals compared to WT mice. Values are *Snai3* transcript per 1000 *Actb* transcripts and are averages from n=3 mice ± standard deviation. * denotes statistically significant reduction in transcript level with a p-values ≤ 0.01.

Figure S5

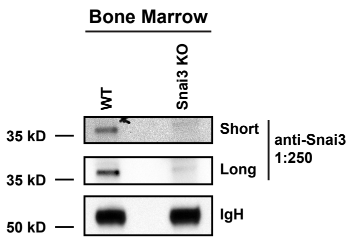

Figure S5. Snai3 immunoblot from WT and *Snai3KO* bone marrow. Whole cell lysates were generated from WT and *Snai3KO* bone marrow. Blots were probed with a Snai3-specific antibody at a dilution of 1:250. Snai1 and Snai2 were not detected. Cross-reactivity to the immunoglobulin heavy chain (IgH) served as an internal loading control. Short and long exposures are shown. Bone marrow from single WT and *Snai3KO* animals were examined.

Figure S6

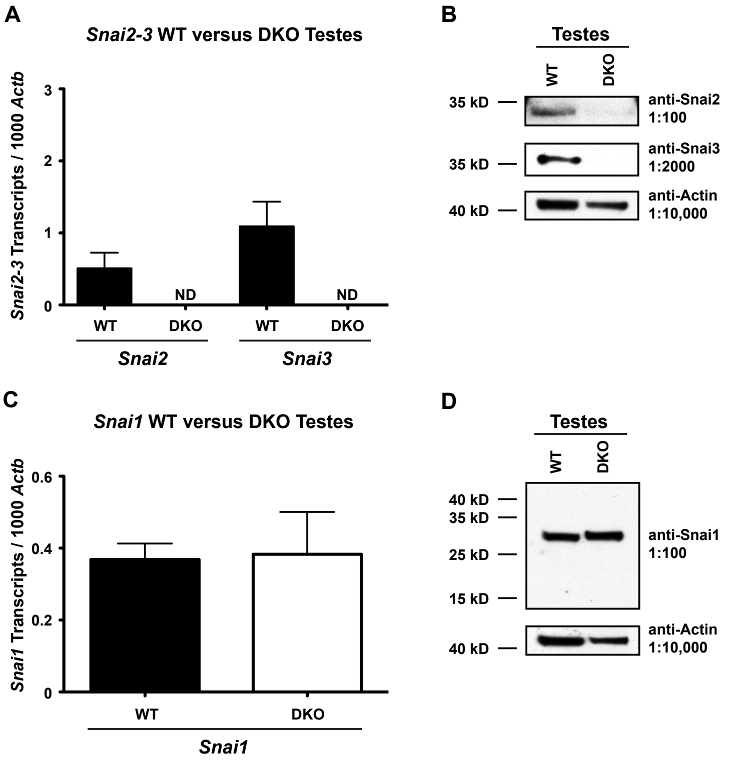

Figure S6. *Snai1, Snai2* and *Snai3* gene expression and protein products in the DKO mice. Testes were harvested from WT and *Snai2/Snai3* DKO animals, and RNA and protein were harvested. (A) Quantitative RT-PCR of RNA obtained from testes for *Snai2* and *Snai3* expression. Data shown are relative transcripts per 1,000 *Actb* transcripts and are averages from n=3 mice, ± standard error (SEM). (B) Immunoblot of Snai2 and Snai3 protein expression in the total testes protein samples with β-actin as loading control. Dilution of antibodies used for the blots is noted. Testes were examined from single WT and DKO animals. (C**)** Quantitative RT-PCR of RNA obtained from testes for *Snai1* expression. Data shown are relative transcripts per 1,000 *Actb* transcripts and are averages from n=3 mice ± standard error (SEM). (D) Immunoblot of Snai1 protein expression in the total testes protein samples with β-actin as loading control. Dilution of the antibodies used for the blots is noted. Testes were examined from single WT and DKO animals.

Figure S7

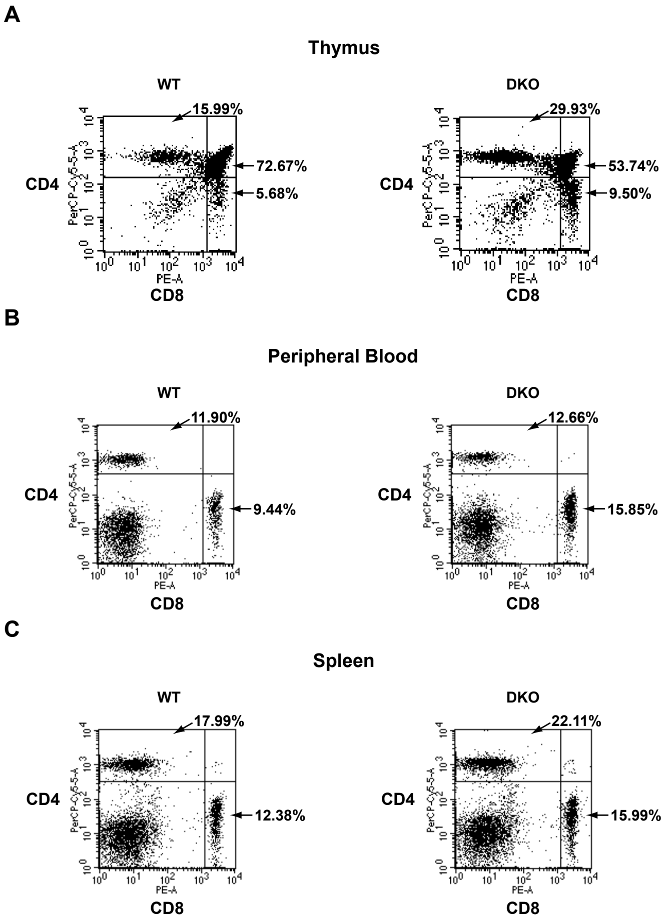

Figure S7. Representative WT and DKO T cell FACS plots. Cells were analyzed for CD4 (y-axis) and CD8 (x-axis) surface expression. In the thymus, double positive cells are represented by $CD4^{+}CD8^{+}$. Representative plots are shown for the thymus (A), peripheral blood (B), and spleen (C), and are from single WT or DKO animals.

Figure S8

A

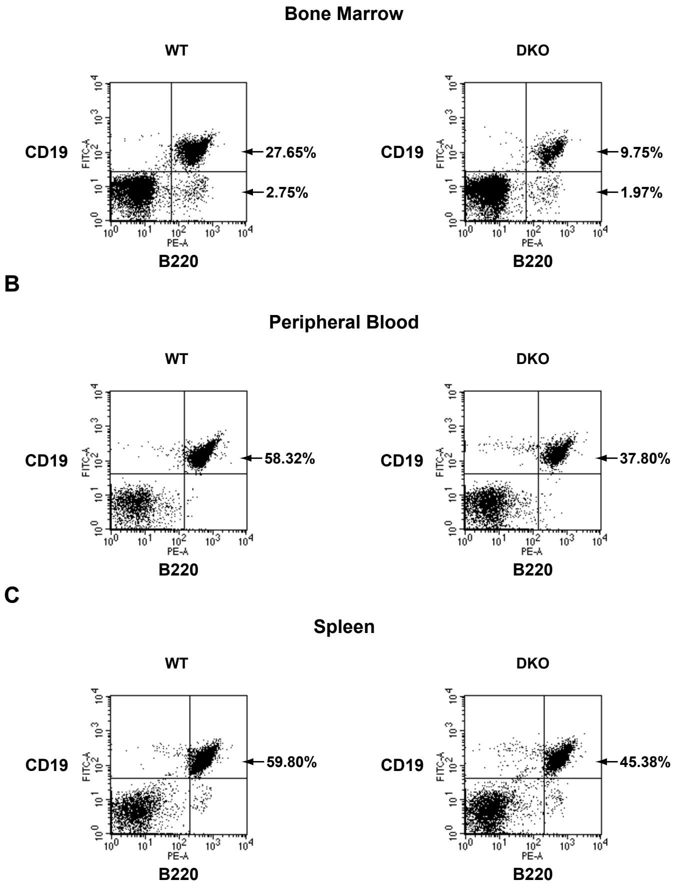

Figure S8. Representative WT and DKO B cell FACS plots. Cells were analyzed for CD19 (y-axis) and B220 (x-axis) surface expression. In bone marrow, $B220^{+}CD19^{-}$ staining represents pre-pro-B cells. $B220^{+}CD19^{+}$ staining represents pro-, pre-, immature, and mature re-circulating B cells. In peripheral blood and spleen, B cells are collectively defined as $B220^{+}CD19^{+}$. Representative plots are shown for the bone marrow (A), peripheral blood (B), and spleen (C), and are from single WT or DKO animals.

Figure S9

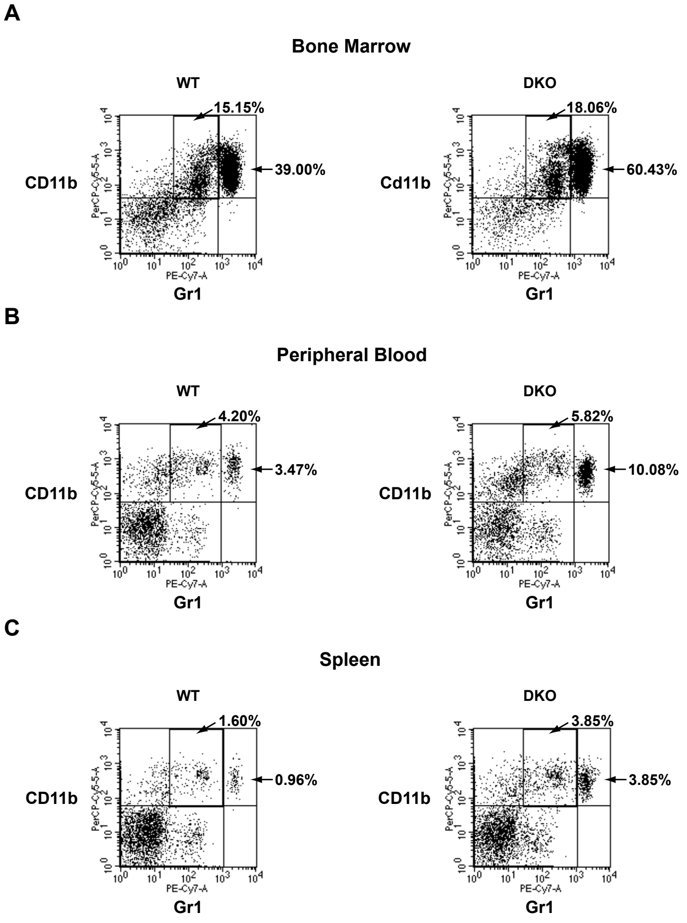

Figure S9. Representative WT and DKO myeloid cell FACS plots. Cells were analyzed for CD11b (y-axis) and Gr1 (x-axis) surface expression. $CD11b^{+}Gr1^{Int}$ staining represents macrophages. $CD11b^{+}Gr1^{Hi}$ staining represents neutrophils, a subset of granulocytes. Representative plots are shown for the bone marrow (A), peripheral blood (B), and spleen (C), and are from single WT or DKO animals.

Table S1: Oligonucleotides used in genotyping and RT-PCR

**Genomic Oligos**	**Forward (5’ -> 3’)**	**Reverse (5’ -> 3’)**
P1 (3093)	ATCTATGCCTGGAAGGGGAGAGG	N/A
P2 (3099)	N/A	GCGTTCACATTCACGGTTGC
P3 (3080)	CCTTGCCAGCACTTGTCTCTTC	N/A
P4 (3079)	N/A	GAAATCTGCCAGCCTCTGCCTATCC
Snai2 WT (4861/4712)	CATCCTTGGGGCGTGTAAGTCC	CACATATTCCTTGTCACAGTACTTGC
Snai2 KO (4709/4707)	CTCTTGGCTGTGATTGGTTACTTC	TGGCGCCTACCGGTGGATGTGGAATG
**RT-PCR Oligos**	**Forward (5’ -> 3’)**	**Reverse (5’ -> 3’)**
Snai1 (4990/4991)	CCGGAAGCCCAACTATAGCG	CGCACTTGGGGTACCAGGAG
Snai2 (4992/4993)	CCTCCAAGAAGCCCAACTAC	GGGTAAAGGAGAGTGGAGTG
Snai3 (QT) (2421/2422)	TGCCGCGCTCCTTCCTGGTG	CAGAGGTACTGTCCCAAGGC
Actb (62/339)	GTAACAATGCCATGTTCAAT	CTCCATCGTGGGCCGCTCTAG

Table S1. Genotyping and RT-PCR Oligos. Primers used for genotyping and RT-PCR analysis. Genomic oligos were used for genotyping. RT-PCR oligos were used for mRNA transcript analysis.
